# The plasma lipids with different fatty acid chains are associated with the risk of hemorrhagic stroke: a Mendelian randomization study

**DOI:** 10.3389/fneur.2024.1432878

**Published:** 2024-07-30

**Authors:** Xingkai Zhang, Xiaoyu Zhu, Qinghai Shi

**Affiliations:** ^1^Department of Graduate School, Xinjiang Medical University, Urumqi, China; ^2^Department of Clinical Laboratory Diagnostic Center, General Hospital of Xinjiang Military Command, Urumqi, China; ^3^School of Medicine, Shihezi University, Shihezi, China

**Keywords:** hemorrhagic stroke, plasma lipids, Mendelian randomization, intracerebral hemorrhage, subarachnoid hemorrhage, PUFA

## Abstract

**Background and objective:**

Hemorrhagic stroke, characterized by acute bleeding due to cerebrovascular lesions, is associated with plasma lipids and endothelial damage. The causal relationship between genetic plasma lipid levels and hemorrhagic stroke remains unclear. This study employs a two-sample Mendelian randomization (MR) analysis to explore the causal relationship between plasma lipid profiles with different fatty acid chains and the risk of intracerebral and subarachnoid hemorrhage, the two main subtypes of hemorrhagic stroke.

**Methods:**

The datasets for exposure and outcome summary statistics were obtained from publicly available sources such as the GWAS Catalog, IEU OpenGWAS project, and FinnGen. The two-sample MR analysis was employed to initially assess the causal relationship between 179 plasma lipid species and the risk of intracerebral and subarachnoid hemorrhage in the Finnish population, leading to the identification of candidate lipids. The same methods were applied to reanalyze data from European populations and conduct a meta-analysis of the candidate lipids. The Inverse Variance Weighting (IVW) method served as the primary analysis for causal inference, with additional methods used for complementary analyses. Sensitivity analysis was conducted to clarify causal relationships and reduce biases.

**Results:**

Two analyses using Mendelian randomization were performed, followed by meta-analyses of the results. A causal relationship was established between 11 specific lipid species and the occurrence of intracerebral hemorrhage within the European population. Additionally, 5 distinct lipid species were associated with subarachnoid hemorrhage. Predominantly, lipids with linoleic acid and arachidonic acid side chains were identified. Notably, lipids containing arachidonic acid chains (C20:4) such as PC 18:1;0_20:4;0 consistently showed a decreased risk of both intracerebral hemorrhage [*p* < 0.001; OR(95% CI) = 0.892(0.835–0.954)] and subarachnoid hemorrhage [*p* = 0.002; OR(95% CI) = 0.794(0.689–0.916)]. Conversely, lipids with linoleic acid chains (C18:2) were associated with an increased risk of intracerebral hemorrhage.

**Conclusion:**

This study identifies a potential causal relationship between lipids with different fatty acid side chains and the risk of intracerebral and subarachnoid hemorrhagic stroke, improving the understanding of the mechanisms behind the onset and progression of hemorrhagic stroke.

## 1 Introduction

Hemorrhagic stroke, marked by acute intracranial bleeding, is clinically significant despite being less common than ischemic stroke. It has a higher mortality rate and more severe clinical outcomes, posing a serious threat to health and quality of life (1). Hemorrhagic stroke encompasses subtypes such as intracerebral hemorrhage (ICH) and subarachnoid hemorrhage (SAH). These subtypes are classified based on bleeding location and have different etiologies (2). Non-traumatic ICH is mainly caused by small vessel diseases like hypertension and arteriosclerosis, leading to ruptured arterioles or microaneurysms (3). In contrast, SAH is typically due to the rupture of large arterial aneurysms or arteriovenous malformations (4). Both ICH and SAH involve endothelial damage and vascular remodeling (2).

Plasma lipids are closely associated with endothelial damage. Dyslipidemia can contribute to endothelial injury and vascular remodeling through various mechanisms, accelerating vascular diseases like atherosclerosis (5). Previous studies have focused on conventional lipid components such as high-density lipoprotein cholesterol (HDL-C), low-density lipoprotein cholesterol (LDL-C), triglycerides (TG), and total cholesterol (TC). Mendelian randomization has been used to examine the impact of these conventional lipids on the incidence of hemorrhagic stroke (6–8).

Modern high-throughput lipidomics technologies have greatly expanded our understanding of the diversity and complexity of circulating lipids. Additionally, genome-wide association studies (GWAS) have transformed our understanding of the genetic variations influencing lipid levels (9, 10). Research shows that while diet affects circulating lipids, plasma levels of these components are heritable, highlighting a significant role of endogenous regulation in lipid metabolism (11). Notably, genetic mechanisms do not uniformly regulate all lipid species within categories.

Exploring the relationship between comprehensive plasma lipid profiles and hemorrhagic stroke development offers insights into early screening and prevention while increasing our understanding of the physiological mechanisms involved. However, a prospective study directly linking plasma lipid profiles to hemorrhagic stroke is still lacking, leaving the causal relationship unclear. Traditional observational studies often have flaws, as data are typically collected post-stroke. Additionally, patients may change their lifestyle or medication after cardiovascular events, altering plasma lipid profiles and further obscuring the causal relationship.

Mendelian randomization (MR) has become increasingly popular for studying disease etiology. In the absence of randomized controlled trials, MR effectively identifies potential disease-causing factors and provides a reliable strategy for investigating causal relationships between exposures and outcomes (12). It determines the causal influence of an exposure on outcomes by using genetic variants associated with the exposure as instrumental variables (IVs), typically derived from single nucleotide polymorphisms (SNPs) (13). Since SNPs are randomly allocated to offspring during conception, confounding factors are significantly reduced, allowing MR to approximate randomized controlled trials to some extent (14). Moreover, MR studies have been employed in stroke risk research to identify various pathogenic factors (15, 16).

There is a significant association between plasma lipid levels and endothelial damage. However, the causal relationship between comprehensive plasma lipid profiles and hemorrhagic stroke remains uncertain. To investigate this, this study will utilize a two-sample MR analysis to evaluate the causal connection between various plasma lipid profiles containing distinct fatty acid side chains and the risk of ICH and SAH. The objective is to provide new strategies and insights for predicting and preventing hemorrhagic stroke.

## 2 Materials and methods

### 2.1 Study design

**Figure 1:**
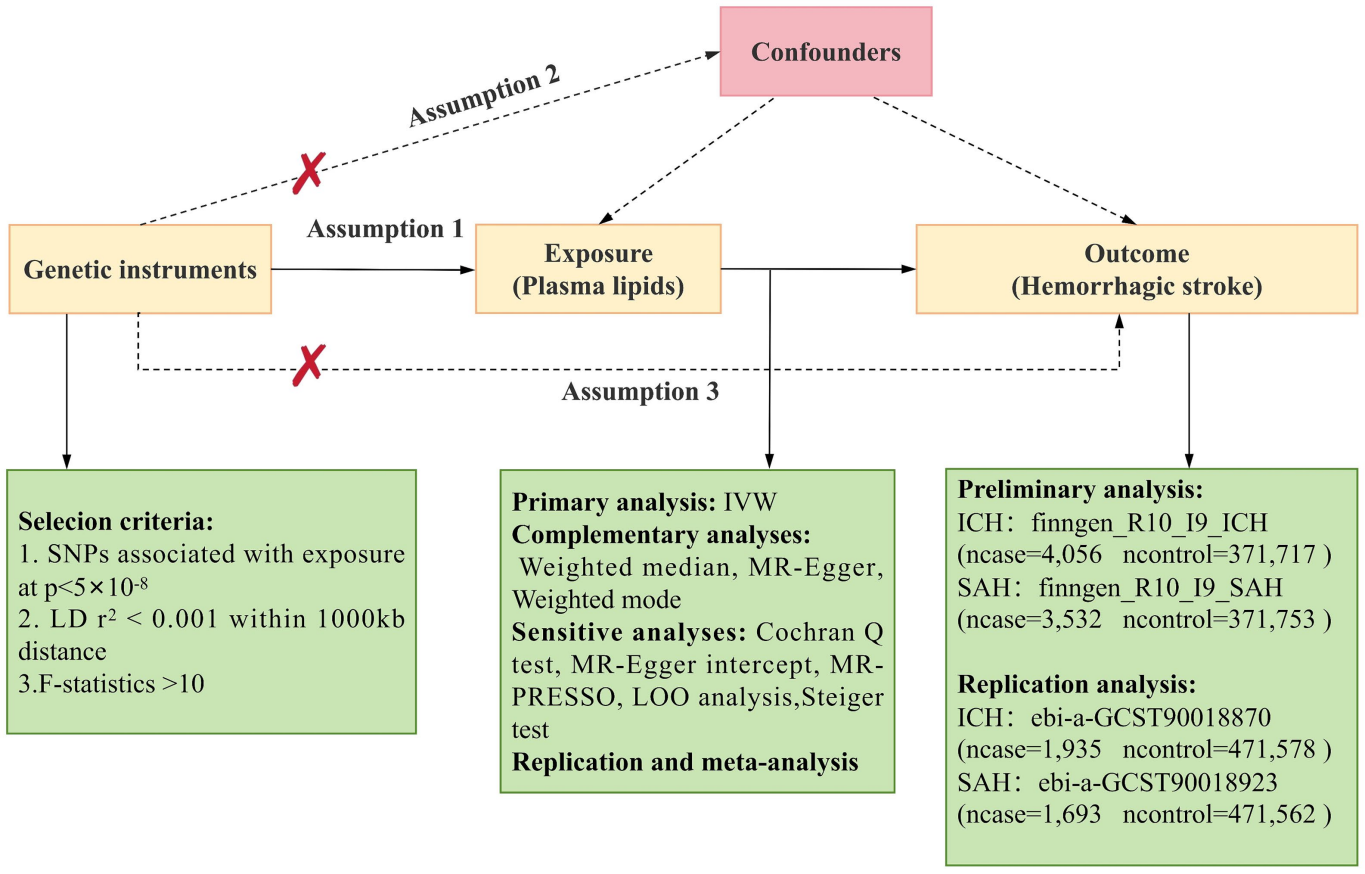
Overview of Mendelian Randomization (MR) analysis. Assumption 1: Genetic instruments are directly and strongly associated with the exposure. Assumption 2: Genetic instruments are independent of potential confounding factors. Assumption 3: Genetic instruments are unrelated to the outcome and only affect the outcome through the exposure. SNPs, single nucleotide polymorphisms; IVW, inverse variance weighted; LD, linkage disequilibrium; LOO analysis, leave-one-out analysis; MR-PRESSO, MR-Pleiotropy RESidual sum and outlier; ICH, Intracerebral hemorrhage; SAH, Subarachnoid Hemorrhage.

Mendelian randomization (MR) employs genetic variations as proxies for risk factors, with effective IVs needing to satisfy three primary assumptions for causal inference (17): (1) Genetic instruments must be directly and strongly associated with the exposure; (2) Genetic instruments should be independent of potential confounding factors; (3) Genetic instruments must be unrelated to the outcome and only influence the outcome through the exposure. The main MR analyses in this study were performed using R software (version 4.2.3) with the Two Sample MR and MR_PRESSO packages. This study’s design was based on the MR study by Yun et al. (18). An overview of the study is presented in Figure 1.

### 2.2 Exposure data sources

The plasma lipids data were sourced from a GWAS conducted by Ottensmann et al., which examined the genetic characteristics of plasma lipidomics (19). This study analyzed genetic variations in 179 lipid species across 13 lipid categories in 7,174 Finnish individuals from the GeneRISK cohort. The aim was to identify genetic variants associated with plasma lipidomic features and assess their impact on certain diseases. The 13 lipid classes included cholesterol (Chol), cholesterol esters (CE), ceramides (CER), diacylglycerols (DAG), lysophosphatidylcholines (LPC), phosphatidylcholines (PC), ether-linked phosphatidylcholines (PCO), phosphatidylethanolamines (PE), lysophosphatidylethanolamines (LPE), ether-linked phosphatidylethanolamines (PEO), sphingomyelins (SM), and triacylglycerols (TAG). These classes follow the naming conventions from Ottensmann et al.’s study. The GWAS data for these 179 lipid species were retrieved from the GWAS Catalog.^1^ Supplementary Table 1 provides detailed information on lipid types, identifiers,^2^ abbreviations, and GWAS numbers.

### 2.3 Outcome data sources

The data for two types of hemorrhagic stroke were initially analyzed using the tenth release of FinnGen^3^ in the Finnish population. FinnGen is a public-private collaboration that integrates genotype data from Finnish biobanks with digital health records from the Finnish National Institute for Health and Welfare. We used the phenotypes labeled “I9_ICH” for ICH, which includes 4,056 cases and 371,717 controls, and “I9_SAH” for SAH, which includes 3,532 cases and 371,753 controls.

For replication analysis, cross-population meta-analysis data on intracerebral and subarachnoid hemorrhages (categorized by ICD-10 codes and phecodes) from Saori’s study were employed, utilizing UK Biobank (UKB) and FinnGen version 3 data (20). The original GWAS data for ICH (ebi-a-GCST90018870, including 1,935 cases and 471,578 controls) and SAH (ebi-a-GCST90018923, including 1,693 cases and 471,562 controls) were obtained from the IEU OpenGWAS project.^4^ Additional information on the GWAS data is available in the study by Saori et al. (20).

### 2.4 Selection of IVs

To meet assumption (1), we refined the criteria for selecting IVs to ensure an accurate and effective assessment of the causal relationship between plasma lipids and disease risk. Initially, only single nucleotide polymorphisms (SNPs) with highly significant associations, specifically those with *p*-values less than 5e-08, were included as IVs for both exposure and outcome. Additionally, to reduce bias from linkage disequilibrium, which can cause a non-random distribution of alleles across multiple genetic loci, we applied stringent screening criteria. These criteria involved selecting SNPs under the conditions of r^2^ = 0.001 and a clumping window of 10,000 kb, ensuring the independence of the IVs and reducing potential biases in subsequent analyses. Only SNPs meeting both the stringent *p*-value threshold and effectively countering the effects of linkage disequilibrium were included in the exposure analysis. Furthermore, to avoid bias from weak instruments, the F statistic was used to assess the correlation strength between each SNP and the exposure. IVs with an F statistic >10 were deemed strong instruments, whereas those with *F* < 10 were considered to have weaker correlations between SNP and exposure. R^2^ and F statistics were calculated as follows (21):


$$R^{2}=\frac{2\times \beta^{2}\times MAF\times \left(1-MAF\right)}{2\times \beta^{2}\times MAF\times \left(1-MAF\right)+2\times {\left(se\left(\beta \right)\right)}^{2}\times N\times MAF\times \left(1-MAF\right)}$$

$$F=\frac{R^{2}}{1-R^{2}}\times \frac{N-k-1}{k}$$

Where β represents the effect size of the target genetic variant, MAF denotes the minor allele frequency of the SNP, and se(β) denotes the standard error of the effect size. R^2^ signifies the proportion of exposure explained by the IVs, or the determination coefficient of the regression equation. N represents the sample size of the exposure, and k indicates the number of SNPs (IVs). To meet assumption (3), SNPs associated with the outcome (*p* < 5e-05) were excluded. Lastly, further MR analysis was conducted on lipids with two or more SNPs.

### 2.5 Statistical analysis

Mendelian randomization (MR) is a technique that employs genetic instruments to investigate causal relationships between modifiable exposures and outcomes. In this study, the Inverse Variance Weighted (IVW) method is used to assess the potential causal effects of plasma lipid profiles on ICH and SAH. The IVW method, preferred for its efficiency with multiple genetic variations as instrumental variables (IVs), calculates the weighted average of the causal effects by integrating the estimates of each genetic variation’s impact on exposure and outcomes (22). Additionally, to reduce the risk of false discoveries from multiple hypothesis testing, the Benjamini and Hochberg false discovery rate (FDR) correction is applied, setting a significance threshold of an FDR-adjusted *p*-value below 0.05 (23). In the IVW analysis, while certain causal associations exhibit *p*-values under 0.05, they do not meet the stricter criterion of an FDR-adjusted *p-*value below 0.05 and are thus regarded as only potentially causal (24).

In this study, in addition to the Inverse Variance Weighted (IVW) method, we utilized three other techniques: Weighted Mode, MR-Egger, and Weighted Median. The Weighted Mode approach addresses correlations between genetic instruments in scenarios similar to those considered by the IVW, which is crucial when using a conservative set of genetic instruments (25). The MR-Egger method conducts weighted regression analysis to estimate the ratios of genetic variation and assesses the average pleiotropic effects through a regression line. It assumes that all genetic variations may exhibit pleiotropic effects, provided these effects are uncorrelated with the genetic variation exposure (26). The Weighted Median technique, by calculating the median of ratio estimates from genetic variations, demonstrates significant resilience against outliers (27).

Sensitivity analyses were conducted to evaluate the presence of horizontal pleiotropy and heterogeneity, which could compromise MR assumptions. Heterogeneity was assessed using Cochran’s Q method, with a *p* < 0.05 indicating significant heterogeneity in the results (28). The MR-Egger intercept test was employed to evaluate horizontal pleiotropy in SNPs used as instrumental variables, with the intercept term suggesting horizontal pleiotropy at *p* < 0.05 (29). Additionally, MR-Pleiotropy RESidual sum and outlier (MR-PRESSO) was utilized to detect horizontally pleiotropic SNPs (30). To ensure the robustness of the results, a leave-one-out (LOO) analysis was performed by sequentially excluding each SNP to determine if any individual SNP significantly influenced the results (31). The Steiger test was also conducted to eliminate biases from reverse causation (25). Detailed characteristics of the relevant analytical methods are provided in Supplementary Table 2.

To confirm the robustness of candidate lipids identified in the initial analysis, a repeat analysis was carried out using an alternative set of GWAS data, detailed in Supplementary Table 3. The meta-analysis was conducted based on a random effects model using Review Manager 5.4.1 software.

## 3 Results

### 3.1 Preliminary analysis of plasma lipids and ICH

**Figure 2:**
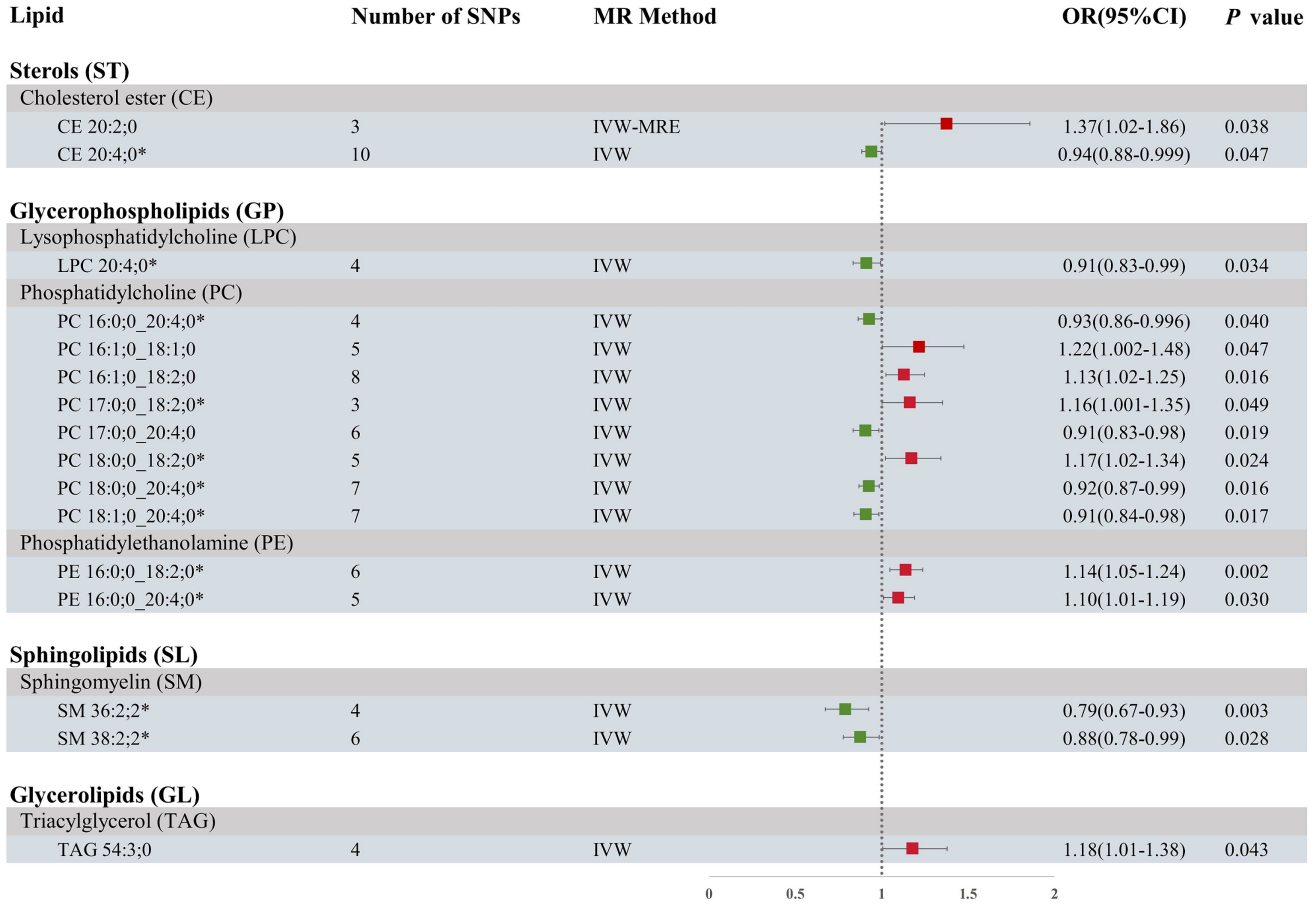
Forest plot of MR results for ICH in the Finnish population, depicting plasma lipids with different fatty acid chains, based on Inverse Variance Weighted (IVW) analysis. SNPs, single nucleotide polymorphisms; IVW, Inverse variance weighted; IVW-MRE, Inverse variance weighted (multiplicative random effects); OR, odds ratio; CI, confidence interval. *represents candidate lipids identified in preliminary analysis.

Our research findings indicate that by employing the IVW method with a significance threshold of *p* < 0.05, we initially identified 16 lipids with potential causal relationships to ICH in the Finnish population (Figure 2). Of the 13 lipid classes analyzed, 6 lipid classes were significantly associated with ICH, with PC showing the strongest relationship. However, after applying FDR correction, none of the results met the significance threshold, implying that the associated lipids may have potential causal relationships. To refine our selection of candidate lipids, we focused on those with significant estimates in IVW (*p* < 0.05) and assessed their consistency in both direction and magnitude across IVW, Weighted Mode, MR-Egger, and Weighted Median methods (Table 1; Supplementary Figure 1). We further ensured that the selected lipids exhibited no heterogeneity via the Cochran Q test (*p* > 0.05) and confirmed the absence of horizontal pleiotropy using the MR-Egger intercept test (*p* > 0.05) and MR-PRESSO results (*p* > 0.05). The Steiger test was then conducted to validate the causal relationships between these lipids and ICH (Table 1). LOO analysis verified that individual SNPs in candidate lipids did not bias the Mendelian Randomization analysis (Supplementary Figure 2). Based on comprehensive sensitivity analyses, we ultimately identified 11 lipids as candidates associated with ICH: CE 20:4;0 [*p* = 0.047; OR(95% CI) = 0.94 (0.88–0.999)], LPC 20:4;0 [*p* = 0.034; OR(95% CI) = 0.91 (0.83–0.99)], PC 16:0;0_20:4;0 [*p* = 0.040; OR(95% CI) = 0.93 (0.86–0.996)], PC 17:0;0_18:2;0 [*p* = 0.049; OR(95% CI) = 1.16 (1.001–1.35)], PC 18:0;0_18:2;0 [*p* = 0.024; OR(95% CI) = 1.17 (1.02–1.34)], PC 18:0;0_20:4;0 [*p* = 0.016; OR(95% CI) = 0.92 (0.87–0.99)], PC 18:1;0_20:4;0 [*p* = 0.017; OR(95% CI) = 0.91 (0.84–0.98)], PE 16:0;0_18:2;0 [*p* = 0.002; OR(95% CI) = 1.14 (1.05–1.24)], PE 16:0;0_20:4;0 [*p* = 0.03; OR(95% CI) = 1.10 (1.01–1.19)], SM 36:2;2 [*p* = 0.003; OR(95% CI) = 0.79 (0.67–0.93)] and SM 38:2;2 [*p* = 0.028; OR(95% CI) = 0.88 (0.78–0.99)].

**Table 1:** Supplementary and sensitivity analysis of causality between plasma lipids and ICH in preliminary analysis.

Lipid		N	MR analysis			Heterogeneity		Pleiotropy		MR-PRESSO		Steiger test	
			Meth	OR (95% CI)	*p*	Q	*p*	Int	*p*	RSS	*p*	*p*	D
Cholesterol ester (CE)													
	CE 20:2;0	3	ME	0.70(0.12–4.12)	0.76	7.21	0.03	0.148	0.59	NA	NA	9.44E-48	T
			WME	1.32(1.09–1.61)	0.01								
			WMO	1.31(1.05–1.65)	0.14								
	CE 20:4;0	10	ME	0.92(0.82–1.02)	0.16	9.06	0.43	0.01	0.6	9.29	0.63	0	T
			WME	0.94(0.88–1.01)	0.08								
			WMO	0.95(0.89–1.01)	0.13								
Lysophosphatidylcholine (LPC)													
	LPC 20:4;0	4	ME	0.99(0.86–1.14)	0.91	3.64	0.30	−0.034	0.28	24.83	0.48	2.37E-227	T
			WME	0.93(0.86–1.01)	0.07								
			WMO	0.93(0.86–1.01)	0.17								
Phosphatidylcholine (PC)													
	PC 16:0;0_20:4;0	4	ME	0.98(0.85–1.14)	0.85	2.93	0.40	−0.028	0.45	11.26	0.53	0	T
			WME	0.93(0.86–1.01)	0.07								
			WMO	0.94(0.87–1.01)	0.21								
	PC 16:1;0_18:1;0	5	ME	0.78(0.49–1.23)	0.36	5.45	0.24	0.071	0.13	7.50	0.34	1.52E-53	T
			WME	1.26(1.03–1.54)	0.03								
			WMO	1.28(1.02–1.62)	0.10								
	PC 16:1;0_18:2;0	8	ME	0.98(0.82–1.18)	0.87	5.21	0.63	0.031	0.13	7.78	0.63	5.00E-159	T
			WME	1.10(0.98–1.23)	0.11								
			WMO	1.09(0.97–1.23)	0.18								
	PC 17:0;0_18:2;0	3	ME	1.03(0.65–1.62)	0.92	0.86	0.65	0.026	0.68	NA	NA	1.31E-67	T
			WME	1.15(0.98–1.34)	0.09								
			WMO	1.14(0.96–1.35)	0.27								
	PC 17:0;0_20:4;0	6	ME	1.02(0.88–1.18)	0.82	5.88	0.32	−0.048	0.14	23.14	0.45	1.76E-276	T
			WME	0.91(0.84–0.99)	0.02								
			WMO	0.93(0.85–1.02)	0.18								
	PC 18:0;0_18:2;0	5	ME	1.21(0.79–1.85)	0.44	1.08	0.9	−0.006	0.88	1.63	0.91	1.86E-82	T
			WME	1.16(0.99–1.37)	0.07								
			WMO	1.20(0.99–1.44)	0.13								
	PC 18:0;0_20:4;0	7	ME	0.95(0.86–1.06)	0.43	6.4	0.38	−0.014	0.51	22.98	0.45	0	T
			WME	0.94(0.88–1.01)	0.07								
			WMO	0.94(0.88–1.01)	0.14								
	PC 18:1;0_20:4;0	7	ME	0.99(0.85–1.15)	0.90	4.58	0.6	−0.032	0.24	9.72	0.56	4.72E-258	T
			WME	0.92(0.84–1.003)	0.06								
			WMO	0.93(0.85–1.02)	0.16								
Phosphatidylethanolamine (PE)													
	PE 16:0;0/18:2;0	6	ME	1.06(0.82–1.39)	0.67	1.43	0.92	0.017	0.63	1.63	0.95	3.05E-183	T
			WME	1.13(1.02–1.25)	0.01								
			WMO	1.13(1.01–1.26)	0.08								
	PE 16:0;0/20:4;0	5	ME	1.19(0.98–1.43)	0.18	1.32	0.86	−0.025	0.43	1.52	0.91	5.67E-227	T
			WME	1.11(1.01–1.21)	0.03								
			WMO	1.11(1.01–1.21)	0.09								
Sphingomyelin (SM)													
	SM 36:2;2	4	ME	0.86(0.49–1.51)	0.65	0.11	0.99	−0.014	0.8	0.16	0.99	1.07E-59	T
			WME	0.79(0.66–0.95)	0.01								
			WMO	0.79(0.63–0.98)	0.13								
	SM 38:2;2	6	ME	0.98(0.68–1.41)	0.92	2.56	0.77	−0.021	0.56	3.75	0.77	6.28E-109	T
			WME	0.88(0.76–1.02)	0.08								
			WMO	0.89(0.75–1.06)	0.24								
Triacylglycerol (TAG)													
	TAG 54:3;0	4	ME	0.86(0.57–1.30)	0.55	2.69	0.44	0.054	0.25	7.56	0.43	9.88E-63	T
			WME	1.24(1.03–1.49)	0.02								
			WMO	1.04(0.84–1.28)	0.77								

N, number of single nucleotide polymorphisms; Meth, methods; ME, MR-Egger; WME, Weighted median; WMO, Weighted mode; CI, confidence interval; OR, odds ratio; Int, MR-Egger intercept; MR-PRESSO, MR-Pleiotropy RESidual sum and outlier; RSS, RSSobs; D, Direction;T, True; NA, Not available.

### 3.2 Preliminary analysis of plasma lipids and SAH

**Figure 3:**
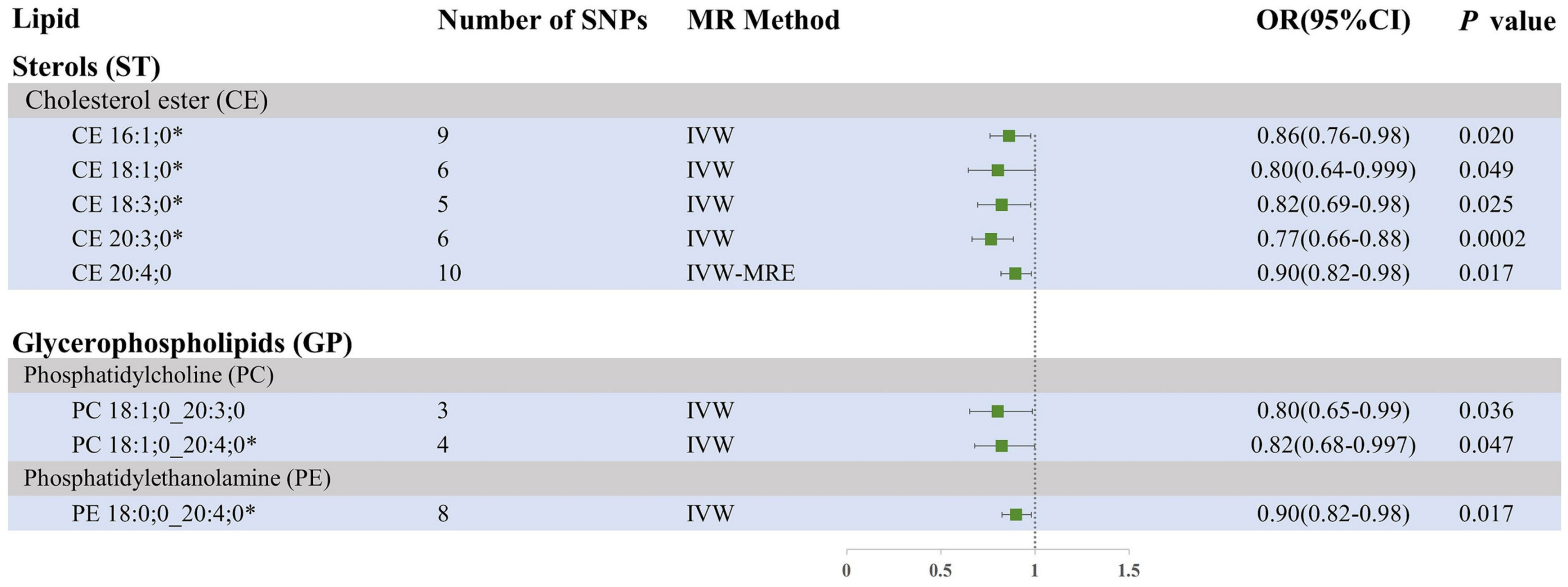
Forest plot of MR results for SAH in the Finnish population, depicting plasma lipids with different fatty acid chains, based on Inverse Variance Weighted (IVW) analysis. SNPs, single nucleotide polymorphisms; IVW, Inverse variance weighted; IVW-MRE, Inverse variance weighted (multiplicative random effects); OR, odds ratio; CI, confidence interval. *represents candidate lipids identified in preliminary analysis.

Our research findings indicate that, using the IVW method with a significance threshold of *p* < 0.05, eight lipids demonstrate potential causal relationships with SAH in the Finnish population (Figure 3). Among the 13 lipid categories analyzed, only three showed significant associations with SAH, with CE demonstrating the strongest causal relationship. However, after applying FDR correction, no lipids met the significance threshold, suggesting potential causal relationships. Consistent with the selection process for candidate lipids in the ICH group, we ensured the consistency of candidate lipid estimates across IVW, Weighted Mode, MR-Egger, and Weighted Median methods (Table 2; Supplementary Figure 3). The MR-PRESSO, Cochran Q test, and MR-Egger intercept test confirmed the absence of heterogeneity and horizontal pleiotropy, while the Steiger test validated the causal relationships between candidate lipids and SAH onset (Table 2). LOO analysis results also indicated no bias (Supplementary Figure 4). Comprehensive sensitivity analyses identified six lipids as potential biomarkers for SAH: CE 16:1;0 [*p* = 0.02; OR (95% CI) = 0.86 (0.76–0.98)], CE 18:1;0 [*p* = 0.049; OR (95% CI) = 0.80 (0.64–0.999)], CE 18:3;0 [*p* = 0.025; OR (95% CI) = 0.82 (0.69–0.98)], CE 20:3;0 [*p* = 0.0002; OR (95% CI) = 0.77 (0.66–0.88)], PC 18:1;0_20:4;0 [*p* = 0.047; OR (95% CI) = 0.82 (0.68–0.997)], and PE 18:0;0_20:4;0 [*p* = 0.017; OR (95% CI) = 0.90 (0.82–0.98)].

**Table 2:** Supplementary and sensitivity analysis of causality between plasma lipids and SAH in preliminary analysis.

Lipid		N	MR analysis			Heterogeneity		Pleiotropy		MR-PRESSO		Steiger test	
			Meth	OR (95% CI)	*p*	Q	*p*	Int	*p*	RSS	*p*	*p*	D
Cholesterol ester (CE)													
	CE 16:1;0	9	ME	0.79(0.55–1.11)	0.22	8.99	0.34	0.023	0.59	11.14	0.38	1.15E-123	T
			WME	0.83(0.71–0.98)	0.03								
			WMO	0.86(0.71–1.05)	0.18								
	CE 18:1;0	6	ME	0.43(0.19–1.01)	0.12	7.36	0.20	0.155	0.21	10.49	0.25	1.58E-51	T
			WME	0.80(0.62–1.02)	0.08								
			WMO	0.77(0.53–1.11)	0.22								
	CE 18:3;0	5	ME	0.64(0.40–1.01)	0.15	3.36	0.50	0.042	0.33	4.29	0.61	4.58E-60	T
			WME	0.82(0.66–1.01)	0.06								
			WMO	0.82(0.66–1.02)	0.15								
	CE 20:3;0	6	ME	0.57(0.34–0.98)	0.11	5.01	0.41	0.057	0.33	7.00	0.48	9.02E-83	T
			WME	0.81(0.69–0.96)	0.02								
			WMO	0.82(0.67–0.999)	0.11								
	CE 20:4;0	10	ME	1.03(0.92–1.15)	0.63	17.44	0.04	−0.06	0.02	58.43	0.28	0	T
			WME	0.93(0.87–1.01)	0.07								
			WMO	0.94(0.88–1.02)	0.15								
Phosphatidylcholine (PC)													
	PC 18:1;0_20:3;0	3	ME	1.07(0.39–2.99)	0.91	0.94	0.63	−0.056	0.67	NA	NA	1.24E-40	T
			WME	0.75(0.59–0.96)	0.02								
			WMO	0.74(0.54–1.02)	0.21								
	PC 18:1;0_20:4;0	4	ME	0.94(0.40–2.21)	0.89	1.07	0.78	−0.018	0.79	1.60	0.84	3.30E-46	T
			WME	0.83(0.66–1.04)	0.11								
			WMO	0.83(0.65–1.06)	0.24								
Phosphatidylethanolamine (PE)													
	PE 18:0;0_20:4;0	8	ME	0.83(0.65–1.06)	0.19	5.40	0.61	0.021	0.54	6.26	0.71	4.59E-245	T
			WME	0.91(0.82–1.02)	0.1								
			WMO	0.92(0.82–1.02)	0.16								

N, number of single nucleotide polymorphisms; Meth, methods; ME, MR-Egger; WME, Weighted median; WMO, Weighted mode; CI, confidence interval; OR, odds ratio; Int, MR-Egger intercept; MR-PRESSO, MR-Pleiotropy RESidual sum and outlier; RSS, RSSobs; D, Direction; T, True; NA, Not available.

### 3.3 Replication and meta-analysis

**Figure 4:**
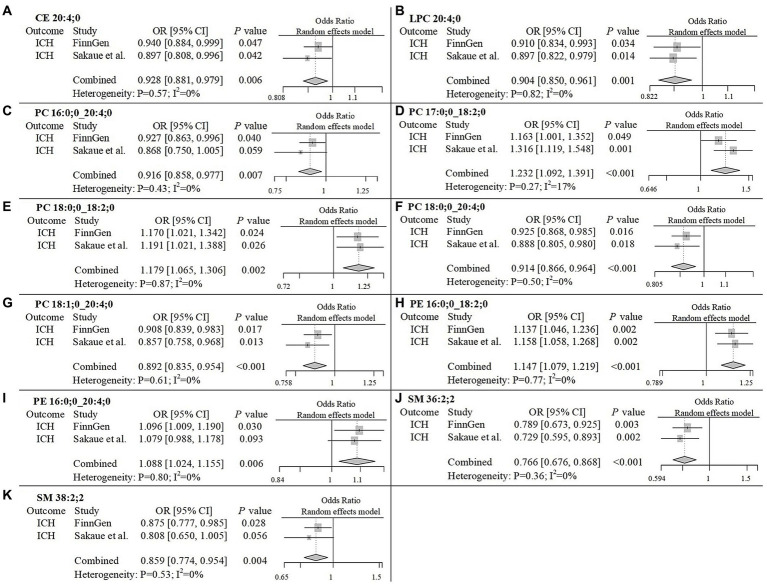
Meta-analysis of significantly associated candidate lipids in ICH. OR, odds ratio; 95%CI, 95% confidence interval.

**Figure 5:**
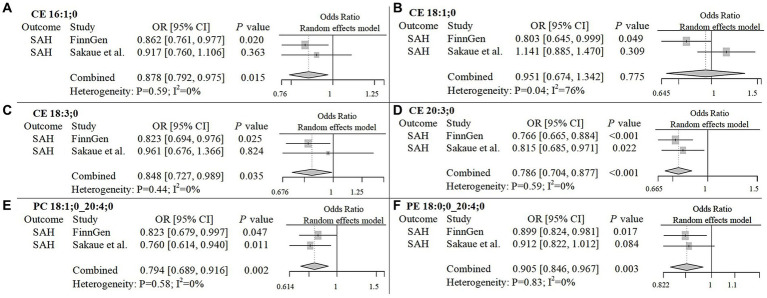
Meta-analysis of significantly associated candidate lipids in SAH. OR, odds ratio; 95%CI, 95% confidence interval.

To improve the reliability of our findings, we replicated the Mendelian Randomization (MR) analysis using GWAS data on ICH and SAH from Saori et al.’s study, which included 179 lipids. The repeated analysis, using the IVW method with a statistical significance threshold of *p* < 0.05, identified causal relationships between 20 lipids and ICH in the European population, and 13 lipids and SAH (Supplementary Figure 5; Supplementary Table 4). From the candidate lipids identified in the initial analysis (Sections 3.1 and 3.2), we performed a meta-analysis to compare the results of the repeated analysis with the initial analysis, further investigating the role of lipids in hemorrhagic stroke in the European population. The meta-analysis results showed that the risk associations of 11 candidate lipids in the ICH group remained consistent (Figure 4). Specifically, elevated levels of CE 20:4;0 [*p* = 0.006; OR(95% CI) = 0.929 (0.881–0.979)], LPC 20:4;0 [*p* = 0.001; OR(95% CI) = 0.904 (0.850–0.961)], PC 16:0;0_20:4;0 [*p* = 0.007; OR(95% CI) = 0.916 (0.858–0.977)], PC 18:0;0_20:4;0 [*p* < 0.001; OR(95% CI) = 0.914 (0.866–0.964)], PC 18:1;0_20:4;0 [*p* < 0.001; OR(95% CI) = 0.892 (0.835–0.954)], SM 36:2;2 [*p* < 0.001; OR(95% CI) = 0.766 (0.676–0.868)] and SM 38:2;2 [*p* = 0.004; OR(95% CI) = 0.859 (0.774–0.954)] were associated with a reduced risk of ICH. Conversely, high levels of PC 17:0;0_18:2;0 [*p* < 0.001; OR(95% CI) = 1.232 (1.092–1.391)], PC 18:0;0_18:2;0 [*p* = 0.002; OR(95% CI) = 1.179 (1.065–1.306)], PE 16:0;0_18:2;0 [*p* < 0.001; OR(95% CI) = 1.147 (1.079–1.219)] and PE 16:0;0_20:4;0 [*p* = 0.006; OR(95% CI) = 1.088 (1.024–1.155)] were associated with an increased risk of ICH. In the SAH group, five candidate lipids exhibited consistent risk associations (Figure 5). Elevated levels of CE 16:1;0 [*p* = 0.015; OR(95% CI) = 0.878 (0.792–0.975)], CE 18:3;0 [*p* = 0.035; OR(95% CI) = 0.848 (0.727–0.989)], CE 20:3;0 [*p* < 0.001; OR(95% CI) = 0.786 (0.704–0.877)], PC 18:1;0_20:4;0 [*p* = 0.002; OR(95% CI) = 0.794 (0.689–0.916)] and PE 18:0;0_20:4;0 [*p* = 0.003; OR(95% CI) = 0.905 (0.846–0.967)] were associated with a reduced risk of SAH. However, in the meta-analysis, CE 18:1;0 [*p* = 0.775; OR (95% CI) = 0.951 (0.674–1.342)] did not reach significance in the SAH group (*p* < 0.05). The most significant lipids identified in the meta-analysis were plasma lipids containing arachidonic acid (C20:4), such as PC 18:1;0_20:4;0, which generally had a risk-reducing effect on ICH and SAH, except for PE 16:0;0_20:4;0. Lipids containing linoleic acid (C18:2) were most prevalent in ICH and showed an increased risk of onset.

## 4 Discussion

Utilizing extensive publicly available genetic data, our study explored the causal relationships between 179 plasma lipid species and two subtypes of hemorrhagic stroke within the European population. Previous MR studies on plasma lipid species have typically provided a broad overview of associated phenotypes without delving into the specific impact of plasma lipid species on hemorrhagic stroke subtypes (11, 19). Our MR analysis examined causal links between lipids with varying fatty acid chains and hemorrhagic stroke. Through stringent inclusion criteria and sensitivity analyses, we identified 11 lipids causally associated with ICH and 5 lipids with a causal relationship to SAH in the European population.

In our analysis, lipids of the same class displayed differing effects on the same disease, likely influenced by the specific fatty acid chains, particularly polyunsaturated fatty acids (PUFAs), except for sphingomyelin (SM). For instance, two PCs, PC 18:0;0_18:2;0 and PC 18:0;0_20:4;0, exhibited differing effects on ICH due to the presence of distinct PUFAs. Conversely, lipids from different classes with the same PUFA chain tended to exhibit similar effects. This is illustrated by CE 20:4;0 and PC 18:1;0_20:4;0, which both demonstrated risk-reducing effects on ICH. This could be due to the hydrolysis of PUFAs in the related lipids. However, an exception was noted with PE 16:0;0_20:4;0, which had an opposite effect on ICH.

In our study, lipids containing arachidonic acid (C20:4) and linoleic acid (C18:2), both omega-6 PUFAs, were found to be the most prevalent. Arachidonic acid (AA), a common omega-6 PUFA, is abundantly present in the phospholipids of cell membranes and is typically hydrolyzed into its free form by phospholipase A2 (32, 33). It plays various physiological roles, influencing the functions of endothelial cells and neurons (34). Lipids containing the AA chain were most abundant in hemorrhagic stroke subtypes and had protective effects, lowering the risk of ICH and SAH. This protective mechanism is possibly due to the reparative effects of hydrolyzed AA on the vascular endothelium. Previous research highlights AA’s role in vascular biology via the cytochrome P450 (CYP) pathway (35), with CYP1B1 being the most prevalent enzyme subtype in brain microvessels (36, 37). Animal studies indicate that CYP1B1 deficiency leads to impaired AA metabolism, reducing cerebral microcirculation and compromising the blood–brain barrier (38, 39). Furthermore, AA supports vascular repair through 11,12-epoxyeicosatrienoic acid, a metabolite of CYP 2 J2, which significantly promotes neovascularization (40). However, PE 16:0;0_20:4;0 had an adverse effect on ICH, possibly due to its susceptibility to lipid peroxidation, leading to ferroptosis (41, 42). While a prospective study in China did not find an effect of AA on ICH (43), previous MR studies have consistently shown that higher AA levels significantly reduce the risk of ICH (44).

An increasing number of epidemiological studies suggest that AA plays a critical role in neuroprotection following hemorrhagic stroke (45, 46). One study reported significantly elevated levels of AA and its metabolites in patients with ICH compared to healthy individuals, indicating a protective response (47). This mechanism involves metabolites from both the cytochrome P450 (CYP) enzyme pathway and the lipoxygenase pathway. Specifically, lipoxin A4, synthesized via the lipoxygenase pathway, inhibits inflammation and cell migration (32). In mouse models, activating the lipoxin A4 receptor has been shown to decrease neuroinflammation after ICH (48). In the cyclooxygenase pathway of prostaglandin synthesis, prostaglandin E2, a derivative of AA, may promote the proliferation of neural stem cells in the adult brain’s subventricular zone following ICH, thus aiding in nervous system repair (45).

Linoleic acid (LA) is an omega-6 PUFA and an essential nutrient necessary for human growth and development, accounting for 1 to 2% of daily energy intake (49). However, excessive intake of LA can lead to various diseases (50). In the United Kingdom, vegetarians have a higher incidence of hemorrhagic stroke compared to meat eaters (51), mainly because LA is predominantly found in vegetable oils. A prospective study in China revealed that elevated blood LA levels increased the risk of ICH (43). Research has indicated that LA may reduce blood LDL-C and platelet aggregation (52), which could increase the risk of hemorrhagic stroke (53). Our studies also found that lipids with esterified LA chains are linked to an increased risk of ICH, likely due to the hydrolysis of these chains. Additionally, LA can increase the expression of adhesion molecules in endothelial cells, promote inflammatory cell migration, and inhibit microvascular dilation, leading to vascular diseases (54, 55). LA exhibits high-affinity binding to lipocalin-2, a proinflammatory adipokine that causes vascular inflammation and endothelial dysfunction in mice (56). It also amplifies TNF-α-induced oxidative stress and inflammatory mediators, which damage the vascular endothelium (57, 58). Although LA can be converted into gamma-linolenic acid and subsequently metabolized into AA, excessive intake results in an accumulation of AA-derived pronociceptive lipid mediators (59, 60). However, a systematic review reported no correlation between LA intake and AA levels in human tissues (61). Our findings suggest that lipids containing esterified AA and LA have differing effects on ICH risk, indicating that the stroke risk associated with esterified LA may not be closely related to AA from a genetic standpoint.

Some research indicates that omega-3 PUFAs may lower the risk of coronary heart disease and ischemic stroke (62, 63). Nevertheless, our study discovered that lipids with esterified omega-3 PUFA side chains do not show a significant correlation with hemorrhagic stroke, except for CE 20:3;0 containing alpha-linolenic acid (ALA), which is relevant to SAH. It has been suggested that a higher dietary intake of ALA may help prevent stroke (64), though a cohort study found that while ALA supplementation was associated with improved overall mortality, it did not significantly affect the risk of coronary heart disease and stroke (65). Additionally, no significant association was observed between plasma phospholipid ALA levels and these conditions. In our study, ALA did not show a significant association with ICH risk. Likewise, other omega-3 PUFAs, such as eicosapentaenoic acid (EPA) and docosahexaenoic acid (DHA), demonstrated no significant causal effects. A meta-analysis of 29 prospective studies on omega-3 PUFAs and stroke incidence rates indicated that EPA and DHA were associated with reduced overall stroke and ischemic stroke risks, but not hemorrhagic stroke, which aligns with our findings (66). However, a case–control study in Korea suggested that low omega-3 PUFAs levels in red blood cells might increase the risk of acute ischemic and hemorrhagic strokes (67). In conclusion, more detailed research is necessary to examine the relationship between omega-3 PUFAs and the risks of ICH and SAH.

This study performed an in-depth analysis of genetic data and hemorrhagic stroke using the Mendelian randomization method. It included extensive genetic data containing SNPs information, combined with thorough MR analysis, effectively removing confounding factors to establish more accurate causal relationships. Multiple tests confirmed the robustness and reliability of the results. However, the study has certain limitations. First, the Mendelian randomization method relies on specific assumptions, such as genetic instrumental variables being unrelated to confounding factors, which may not always be valid. Second, most of the genetic data originate from European populations, which may not fully represent the genetic diversity of the broader human population. Finally, the study cannot provide precise individual risk predictions but can only offer general risk trends.

## 5 Conclusion

This study identified seven lipids that may be causally associated with an increased risk of ICH in European populations, and four lipids that may be causally associated with a reduced risk of ICH. Additionally, five lipids were found to be potentially causally associated with a reduced risk of SAH. However, no lipids were found to be associated with an increased risk of SAH. The effects of these plasma lipids on these diseases may be related to the lipids themselves, but are more likely associated with the unsaturated fatty acid side chains they carry. Plasma lipids with arachidonic acid side chains showed a risk-lowering effect on both intracerebral and subarachnoid hemorrhages, while those with linoleic acid side chains were associated only with an increased risk of intracerebral hemorrhage. These findings could contribute to the understanding of the mechanisms behind the onset and progression of hemorrhagic stroke.

## Data availability statement

The original contributions presented in the study are included in the article/Supplementary material, further inquiries can be directed to the corresponding author.

## Ethics statement

Ethical approval was not required for the study involving humans in accordance with the local legislation and institutional requirements. Written informed consent to participate in this study was not required from the participants or the participants’ legal guardians/next of kin in accordance with the national legislation and the institutional requirements.

## Author contributions

XkZ: Writing – review & editing, Writing – original draft, Methodology, Investigation, Data curation. XyZ: Writing – original draft, Writing – review & editing, Visualization. QS: Writing – review & editing, Supervision, Resources, Project administration, Funding acquisition.

## Footnotes

^1^ https://www.ebi.ac.uk/gwas/

^2^ http://www.swisslipids.org

^3^ https://www.finngen.fi/en/access_results

^4^ https://gwas.mrcieu.ac.uk/
