# Supplementary material for: The plasma lipids with different fatty acid chains are associated with the risk of hemorrhagic stroke: a Mendelian randomization study
Source: Front Neurol. 2024 Jul 30;15:1432878. doi: 10.3389/fneur.2024.1432878 (PMC11319180; doi:10.3389/fneur.2024.1432878)
Supplement: Supplementary file 2 [file Table_2.DOCX]

**Supplementary Table 2.** Details the characteristics of the relevant analytical methods.

| **Method** | **Purpose** | **Characteristic** |
| --- | --- | --- |
| Inverse Variance Weighted (IVW)(1,2) | MR analysis | IVW, commonly used in most MR analyses, based on the effect estimate of each instrumental variable and its variance, weights are assigned to the effect estimates, giving greater weight to instrumental variables with smaller variance. This weighting mechanism enhances the efficiency and accuracy of estimates when multiple instrumental variables are present. However, it requires high standards for instrumental variables: they must be associated with the outcome solely through the exposure factor (no genetic confounding), and there should be no horizontal pleiotropy among the instrumental variables. |
| MR-Egger(3,4) | MR analysis | MR Egger addresses the issue of horizontal pleiotropy in genetic instruments by allowing the regression intercept to be non-zero. This approach enables the method to provide unbiased causal estimates even if some SNPs affect the outcome variable through biological pathways not involving the exposure. Unlike the IVW method, MR Egger does not rely on the strict assumption of no horizontal pleiotropy. Instead, it depends on the 'Instrument Strength Independent of Direct Effect' (InSIDE) assumption, which posits that the strength of the instrumental variables is unrelated to their direct effects on the outcome, a condition that is more relaxed than the no pleiotropy assumption. MR Egger can serve as a sensitivity analysis tool for assessing the horizontal pleiotropy of instrumental variables. However, its statistical efficiency is generally lower than that of the IVW method due to larger standard errors in its estimates. Therefore, MR Egger is used only as an auxiliary analysis method in our study. |
| Weighted Median(5) | MR analysis | The weighted median estimator is based on the statistical concept of the median. It calculates the impact of all instrumental variables (SNPs) on the exposure and identifies the median of these effects as the final causal effect estimate. The weighted median estimator assigns different weights to the estimates of each instrumental variable based on the inverse variance of the association strength between each SNP and the outcome variable. This weighting ensures that SNPs providing more precise estimates have a greater influence on the overall estimate.It provides a robust causal effect estimate, even with up to 50% invalid instrumental variables. Its relatively relaxed data requirements allow it to disregard errors such as horizontal pleiotropy in the instrumental variables, resulting in larger standard errors. Therefore, it is used only as an auxiliary analysis method in our study. |
| Weighted Mode(6) | MR analysis | The basic idea of the weighted mode estimator is to select the most frequently occurring pattern (or central tendency) in causal effect estimation. By weighting each genetic instrument's Wald estimate—typically proportional to the precision of the genetic instruments, usually measured by the reciprocal of their standard error—the most common causal effect estimate is identified. The core advantage of the weighted mode estimator is its robustness to invalid instrumental variables (i.e., genetic variations that may not satisfy all MR assumptions). This method identifies and uses the instrumental variables with the most significant impact on the causal estimate, providing a robust causal estimate when the majority of instrumental variables are valid. Compared to the IVW method, the weighted mode estimator is less sensitive to the influence of a single or a few invalid instrumental variables. Even if some instrumental variables are biased, as long as most point to the same causal effect, the weighted mode can still provide the correct direction and estimate. Although the weighted mode is robust to some violations of MR assumptions, if the proportion of invalid instrumental variables exceeds half, the effectiveness and accuracy of this method may be compromised. Therefore, it is used only as an auxiliary analysis method in our study. |
| Cochran's Q test(7) | sensitivity analysis --heterogeneity | Cochran's Q test is used to detect statistical heterogeneity among the Wald estimates of different genetic instrumental variables. Significant heterogeneity may indicate that some genetic variations affect the outcome through different mechanisms or that they do not meet the assumptions of valid instrumental variables for MR analysis. A p-value of less than 0.05 indicates significant heterogeneity, which we do not permit among the instrumental variables for the candidate lipids identified. |
| MR-Egger intercept test(8) | sensitivity analysis --pleiotropy | The instrumental variables for the candidate lipids we select need to show no horizontal pleiotropy in the initial analysis, and the MR-Egger intercept test is one method to detect such pleiotropy. The main purpose of the MR-Egger intercept test is to detect the presence of horizontal pleiotropy bias. The MR-Egger method is similar to adding an intercept term in regression analysis, where the intercept (MR-Egger intercept) indicates whether there is systematic pleiotropy bias. If the intercept is significantly non-zero, it suggests that the genetic instrumental variables may affect the outcome not only through the exposure but also through other unmeasured pathways. If the MR-Egger intercept is significantly non-zero (p < 0.05), it indicates that the MR analysis results may be influenced by horizontal pleiotropy bias, potentially biasing the causal effect estimates. In such cases, relying solely on traditional MR methods like Inverse Variance Weighted (IVW) may not suffice to accurately estimate the causal relationships. |
| MR-Pleiotropy RESidual sum and outlier (MR-PRESSO)(9) | sensitivity analysis --pleiotropy | MR-PRESSO operates by evaluating the residual sum of squares of genetic instruments within MR analyses to detect the presence of horizontal pleiotropy. It identifies outliers by comparing the observed effects of each genetic instrument with the effects predicted by all other instruments. If the effect of a genetic instrument significantly deviates from its expected effect, it may indicate that the instrument is influenced by horizontal pleiotropy. By systematically detecting and correcting for pleiotropy outliers, MR-PRESSO helps researchers obtain more robust and credible estimates of causal relationships. However, MR-PRESSO requires that there be more than 3 SNPs to be utilized. Our study mandates that the candidate lipids in the initial analysis must pass these tests for horizontal pleiotropy (p > 0.05). |
| leave-one-out (LOO) analysis(10) | sensitivity analysis | The leave-one-out (LOO) analysis method increases the credibility of the results by sequentially excluding each genetic instrument (SNP) and redoing the MR analysis with the remaining genetic instruments. If the removal of any single genetic instrument does not significantly change the estimate of the causal effect, it indicates that the MR results do not rely on any single genetic variation. |
| Steiger directionality test(6) | sensitivity analysis | The Steiger directionality test is an important sensitivity analysis in bidirectional Mendelian randomization, given the potential for reverse causality. It is used to ascertain the directionality of a putative MR association, even when the underlying biology of the instrument variants is not understood.While Steiger filtering performed during instrument harmonization compares the variance explained by individual SNPs for the exposure and outcome datasets, removing SNPs if they explain more variance in the outcome than in the exposure, the Steiger directionality test statistically assesses whether the assumption that the exposure causes the outcome is valid. This test compares the estimated variance explained in the exposure to the estimated variance explained in the outcome for all SNPs included in the MR analysis, testing whether the variance in the outcome is less than the variance in the exposure. We set a Steiger directionality test p-value threshold of 0.05 to assess whether the inferred directionality of the MR analysis is true (i.e., p < 0.05 suggests a true causal direction). |

**REFERENCES**

1. Hemani G, Zheng J, Elsworth B, Wade KH, Haberland V, Baird D, et al. The MR-Base platform supports systematic causal inference across the human phenome. *Elife* (2018) **7**. doi:10.7554/eLife.34408

2. Wootton RE, Jones HJ, Sallis HM. Mendelian randomisation for psychiatry: how does it work, and what can it tell us? *Mol Psychiatry* (2022) **27**: 53-57. doi:10.1038/s41380-021-01173-3

3. Bowden J, Davey SG, Burgess S. Mendelian randomization with invalid instruments: effect estimation and bias detection through Egger regression. *Int J Epidemiol* (2015) **44**: 512-25. doi:10.1093/ije/dyv080

4. Bowden J, Del GMF, Minelli C, Davey SG, Sheehan NA, Thompson JR. Assessing the suitability of summary data for two-sample Mendelian randomization analyses using MR-Egger regression: the role of the I2 statistic. *Int J Epidemiol* (2016) **45**: 1961-74. doi:10.1093/ije/dyw220

5. Bowden J, Davey SG, Haycock PC, Burgess S. Consistent Estimation in Mendelian Randomization with Some Invalid Instruments Using a Weighted Median Estimator. *Genet Epidemiol* (2016) **40**: 304-14. doi:10.1002/gepi.21965

6. Hemani G, Tilling K, Davey SG. Correction: Orienting the causal relationship between imprecisely measured traits using GWAS summary data. *Plos Genet* (2017) **13**: e1007149. doi:10.1371/journal.pgen.1007149

7. Bowden J, Del GMF, Minelli C, Zhao Q, Lawlor DA, Sheehan NA, et al. Improving the accuracy of two-sample summary-data Mendelian randomization: moving beyond the NOME assumption. *Int J Epidemiol* (2019) **48**: 728-42. doi:10.1093/ije/dyy258

8. Rees J, Wood AM, Burgess S. Extending the MR-Egger method for multivariable Mendelian randomization to correct for both measured and unmeasured pleiotropy. *Stat Med* (2017) **36**: 4705-18. doi:10.1002/sim.7492

9. Verbanck M, Chen CY, Neale B, Do R. Detection of widespread horizontal pleiotropy in causal relationships inferred from Mendelian randomization between complex traits and diseases. *Nat Genet* (2018) **50**: 693-98. doi:10.1038/s41588-018-0099-7

10. Burgess S, Thompson SG. Interpreting findings from Mendelian randomization using the MR-Egger method. *Eur J Epidemiol* (2017) **32**: 377-89. doi:10.1007/s10654-017-0255-x
