# Supplementary material for: The plasma lipids with different fatty acid chains are associated with the risk of hemorrhagic stroke: a Mendelian randomization study
Source: Front Neurol. 2024 Jul 30;15:1432878. doi: 10.3389/fneur.2024.1432878 (PMC11319180; doi:10.3389/fneur.2024.1432878)

**Supplementary Figure 1.** Scatterplot of significantly associated candidate lipids with consistent directionality for the association between plasma lipids and intracerebral hemorrhage.


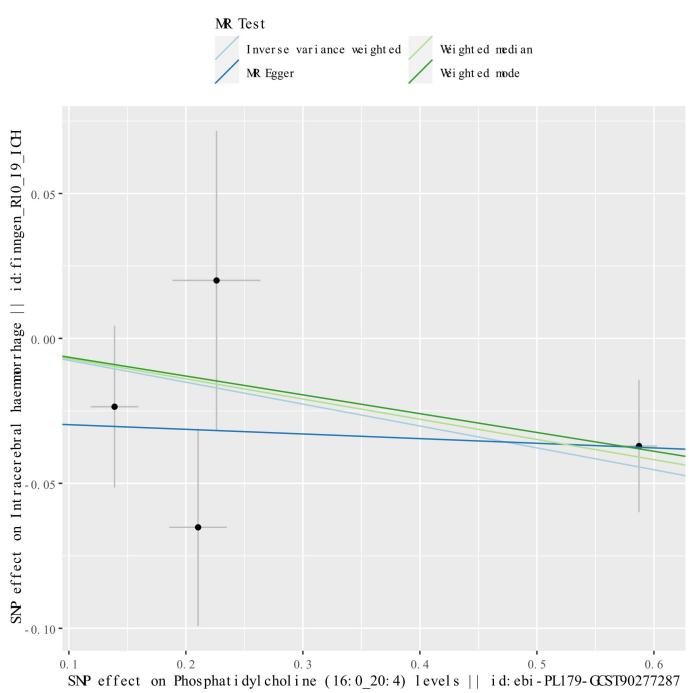

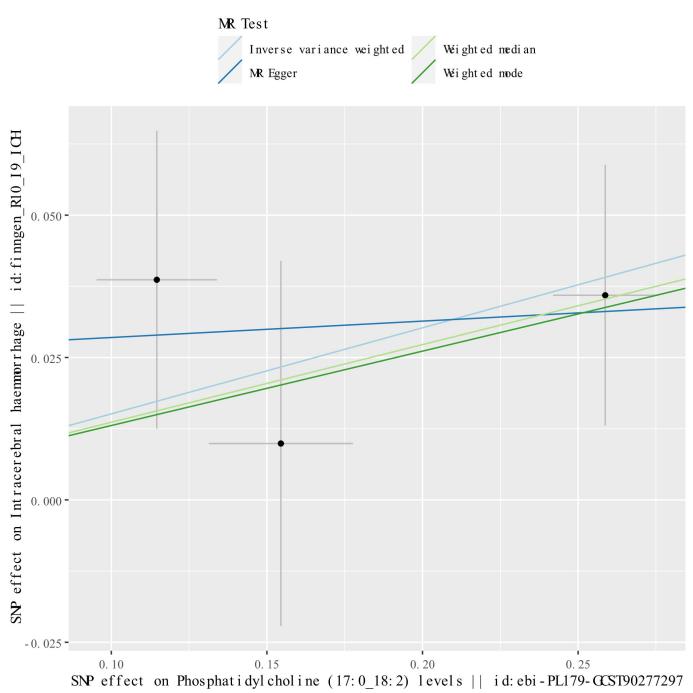

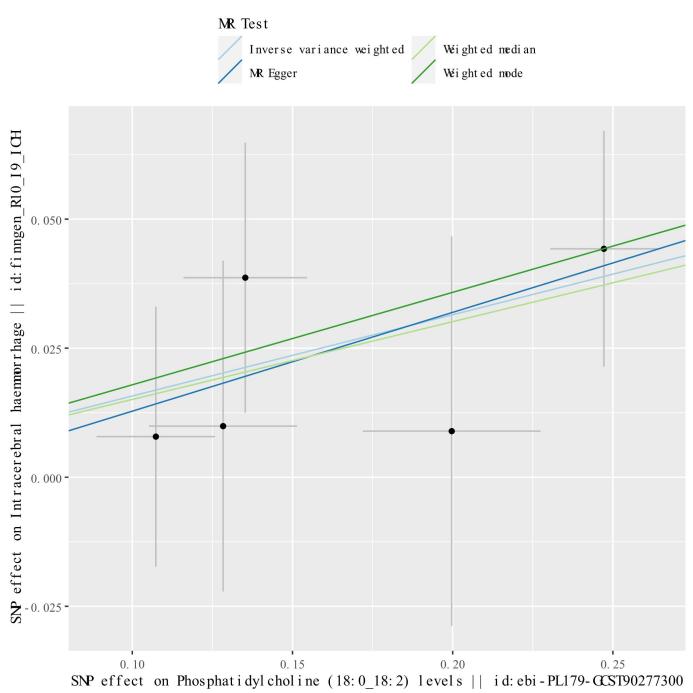

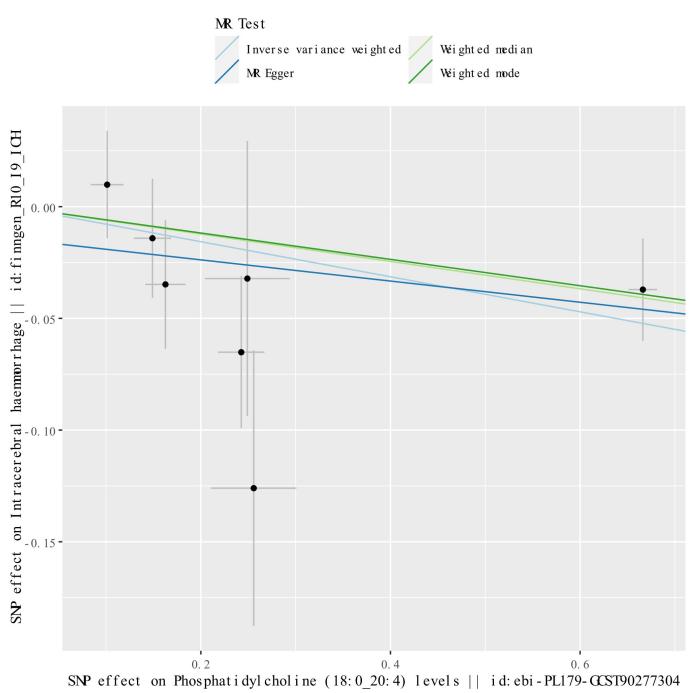

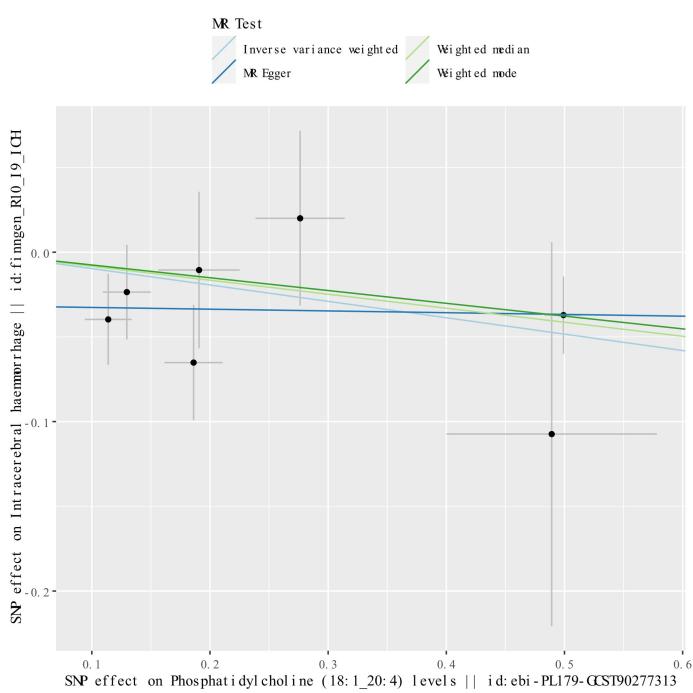
8
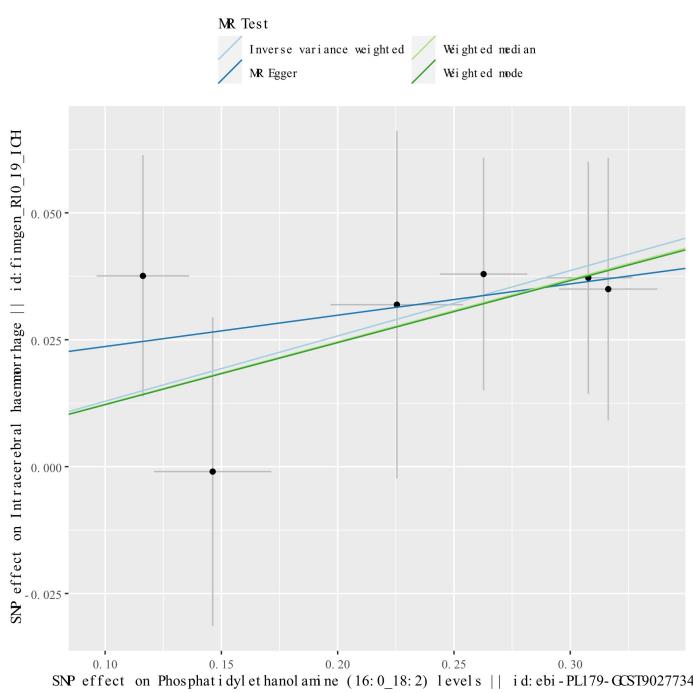

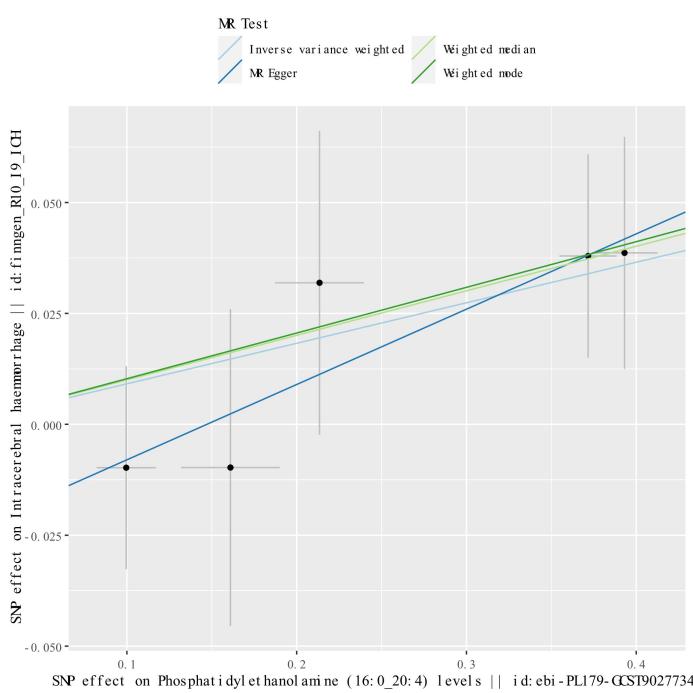

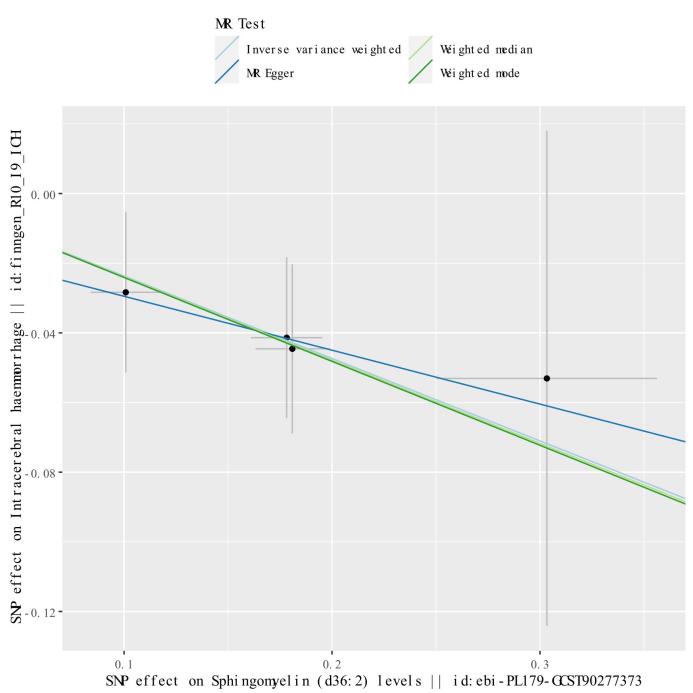

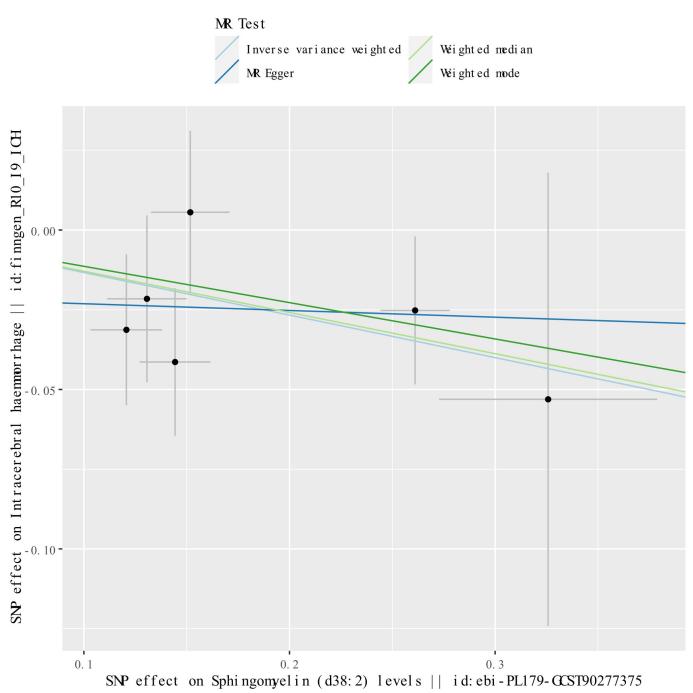

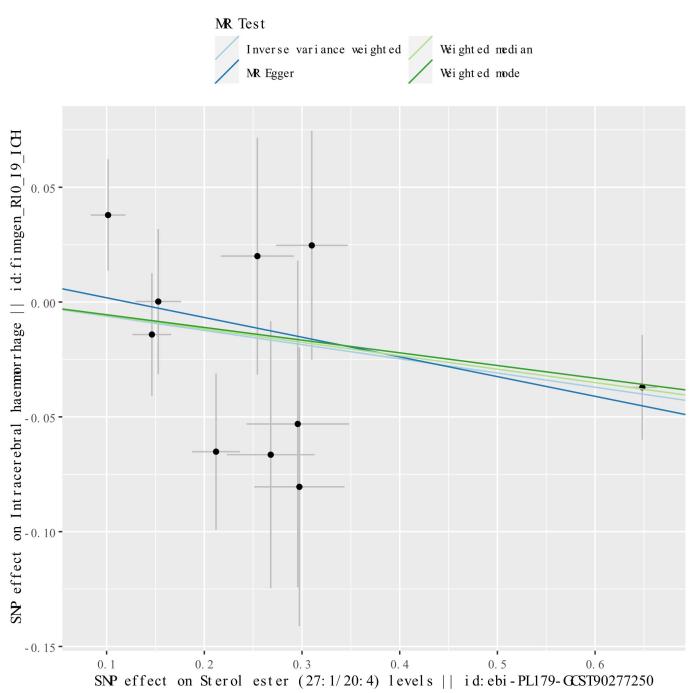

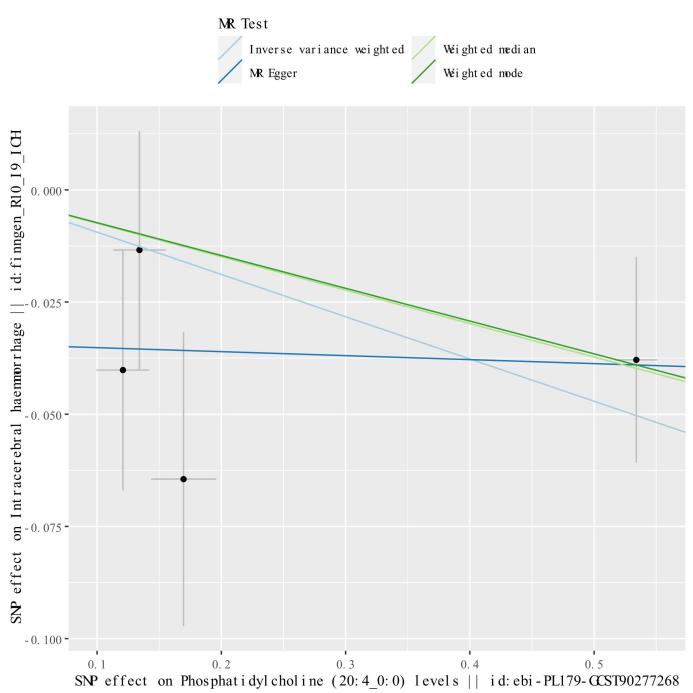

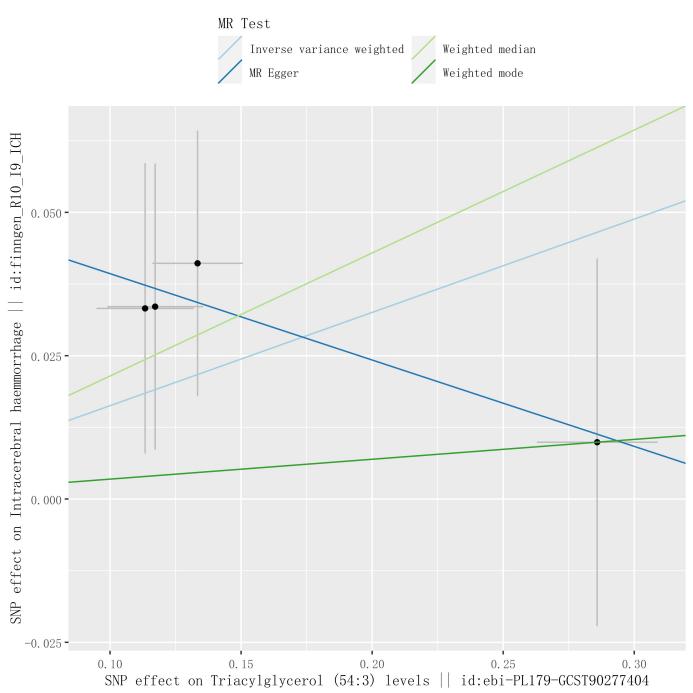

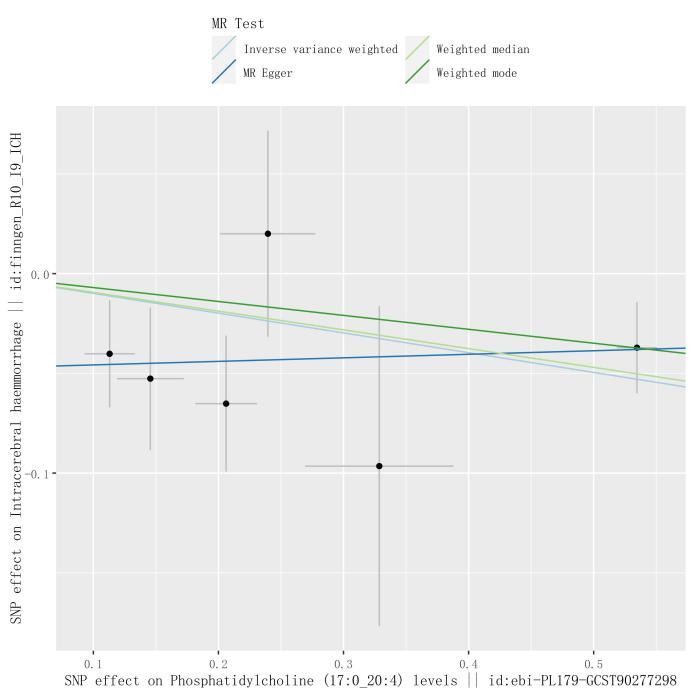

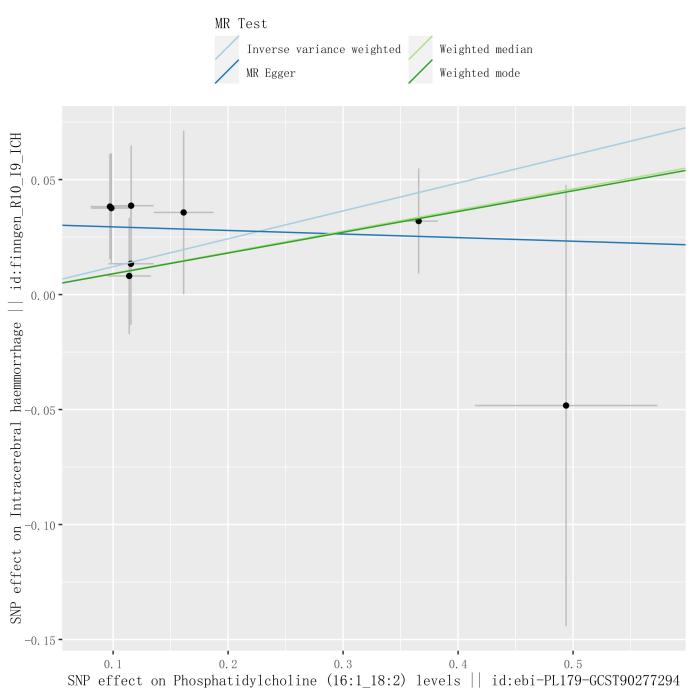

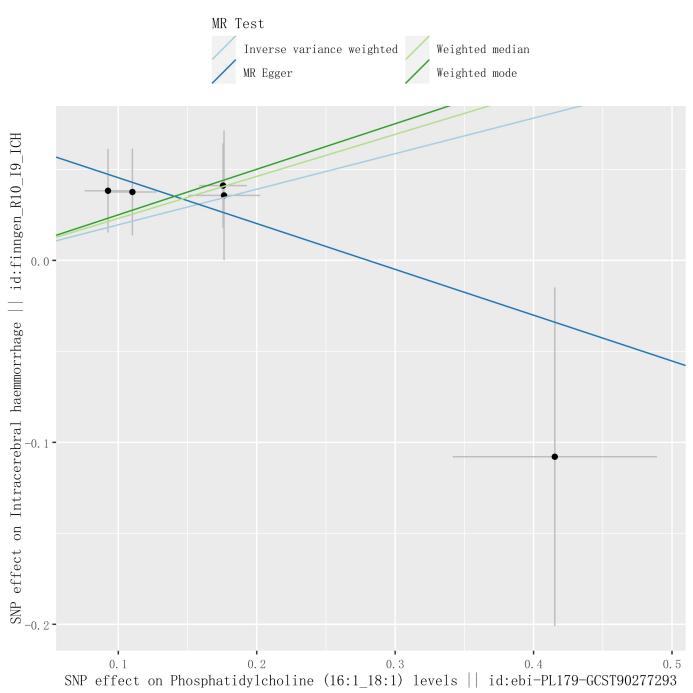

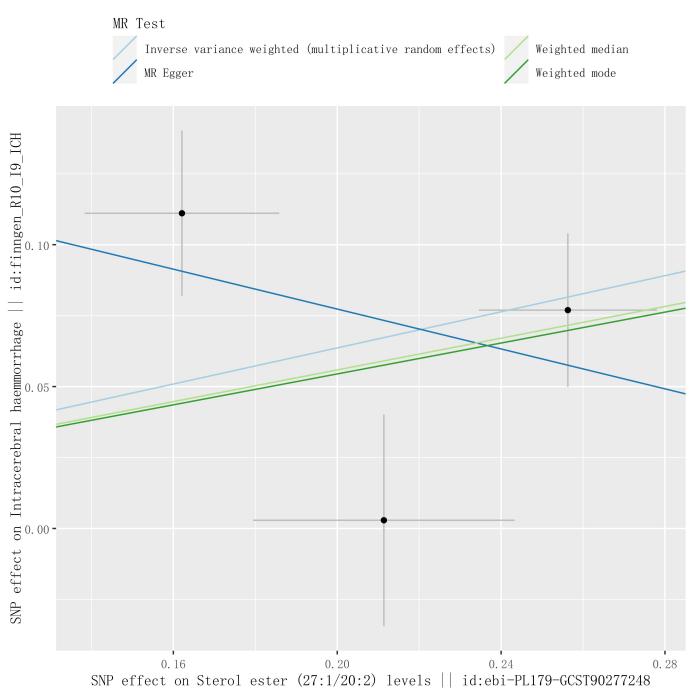


**Supplementary Figure 2.** Forest plot of leave-one-out analysis in Mendelian randomization (MR) with significant inverse variance weighted (IVW) estimates for the association between plasma lipids and intracerebral hemorrhage.


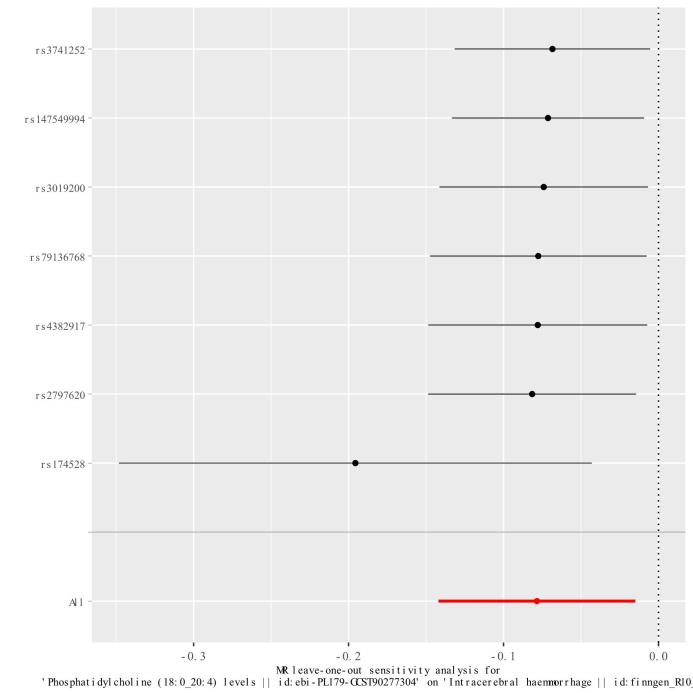

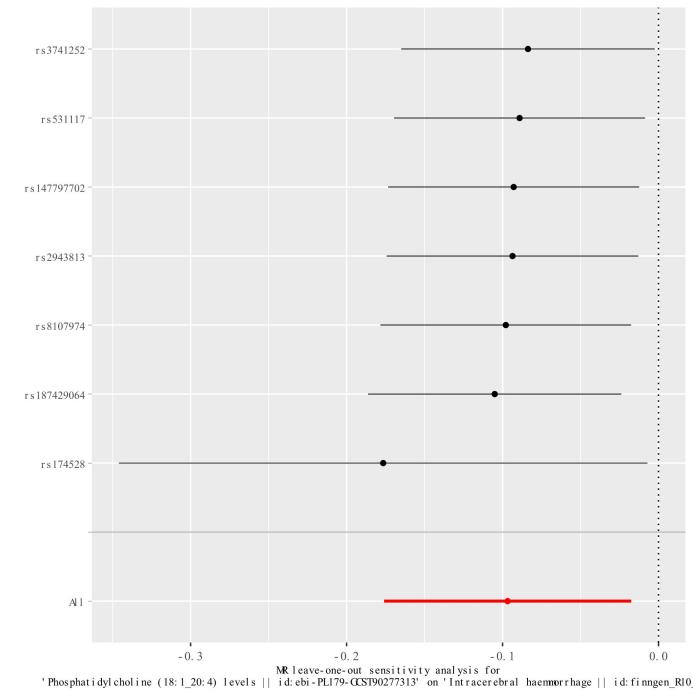

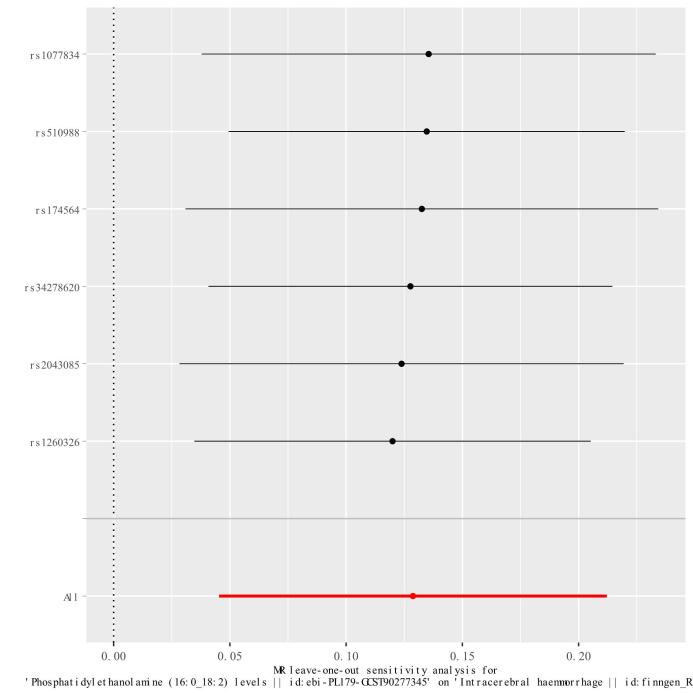

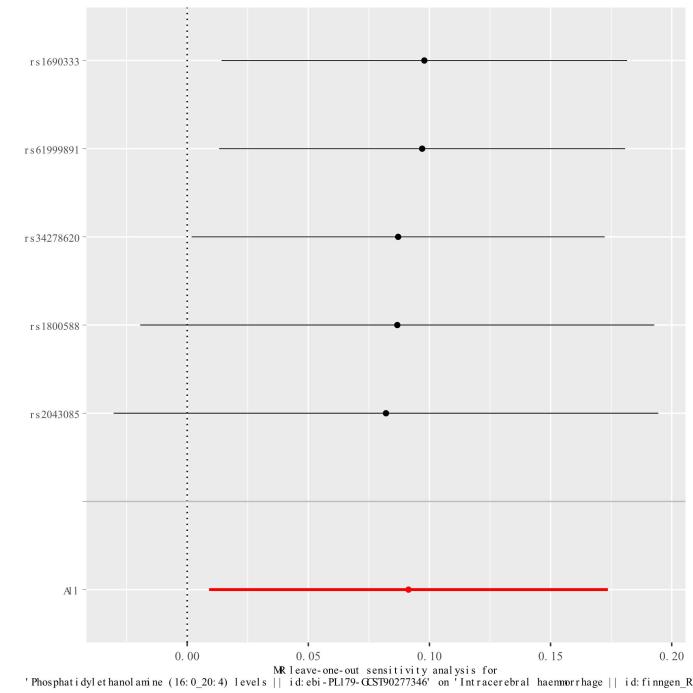

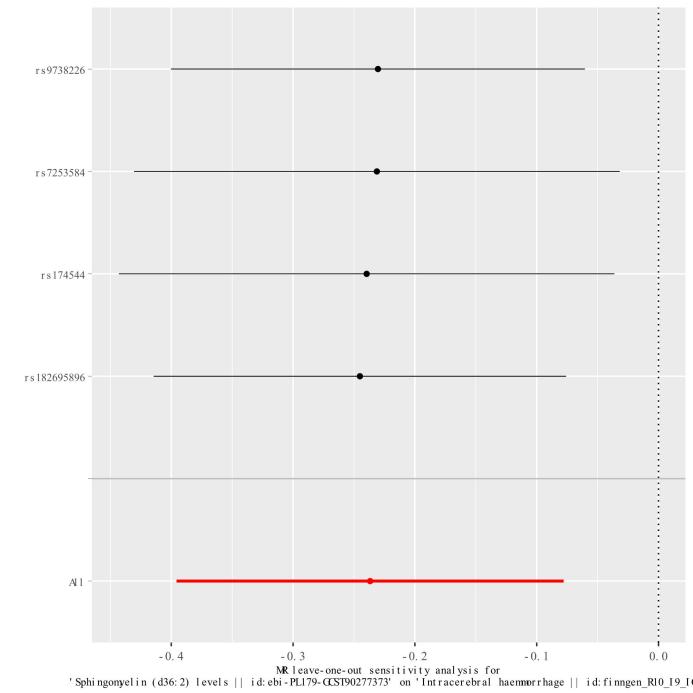

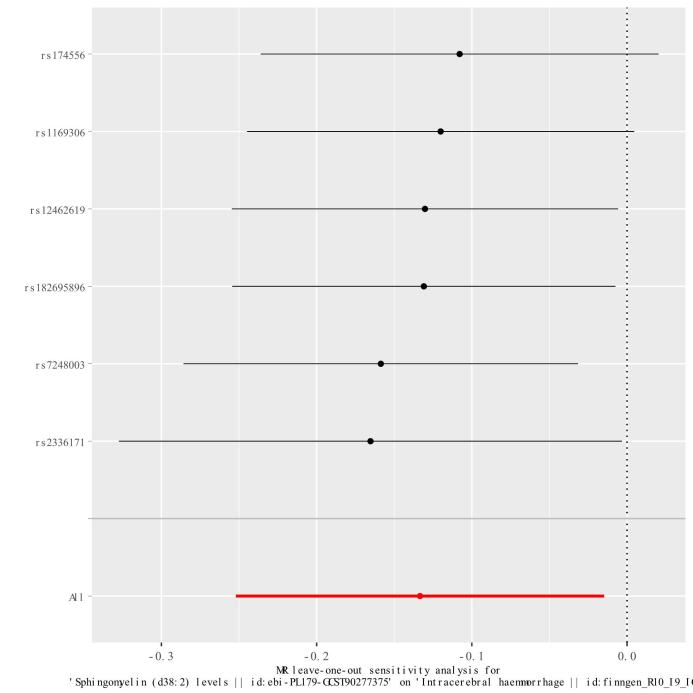

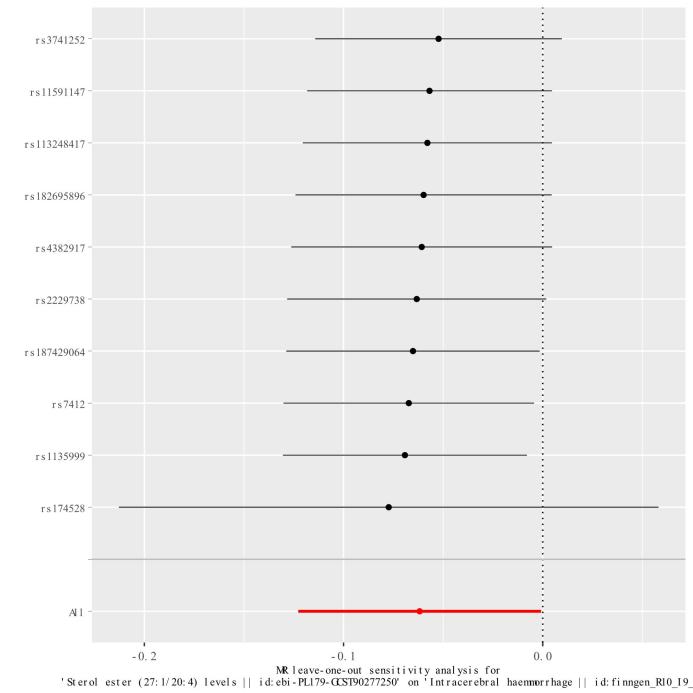

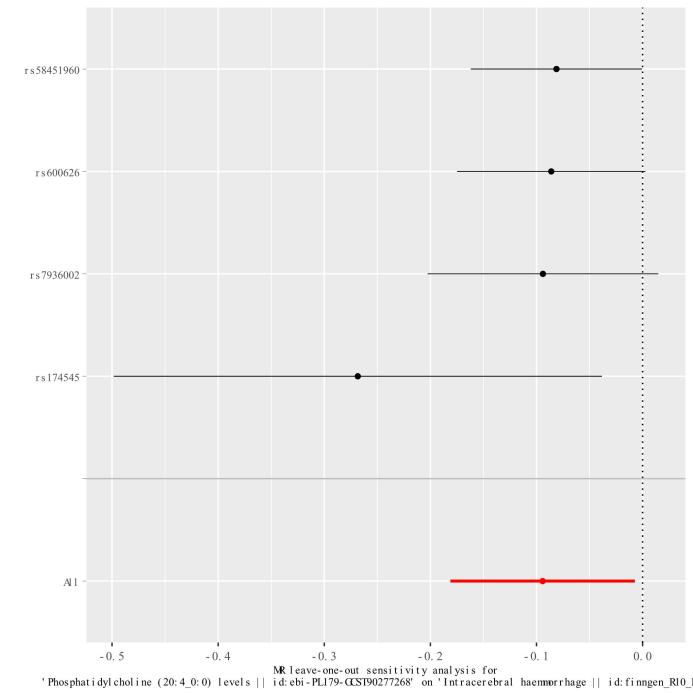

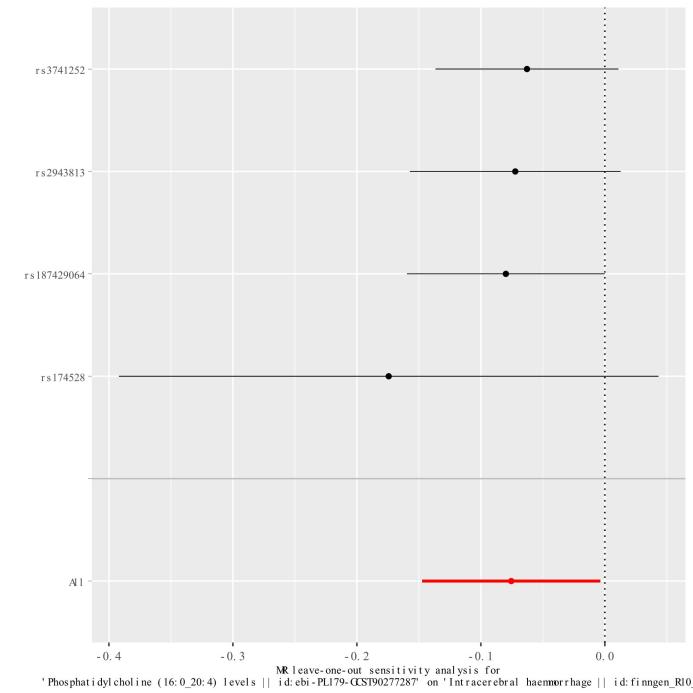

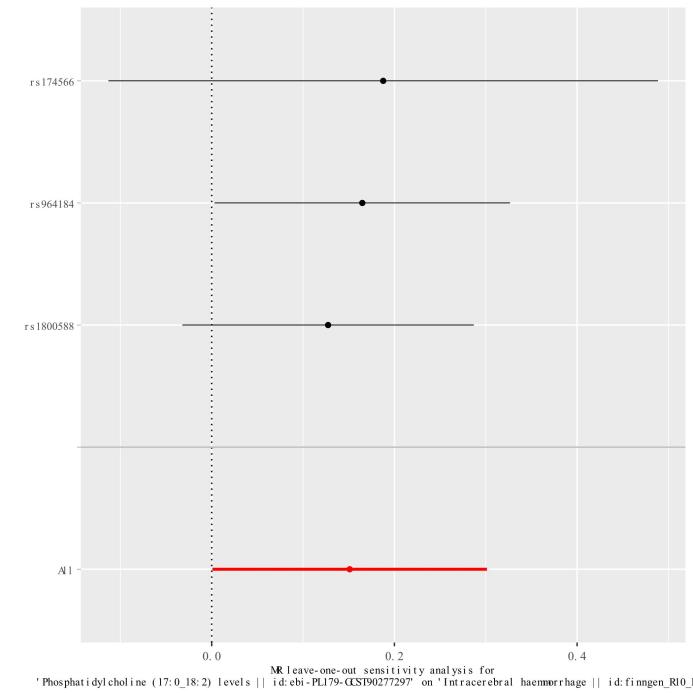

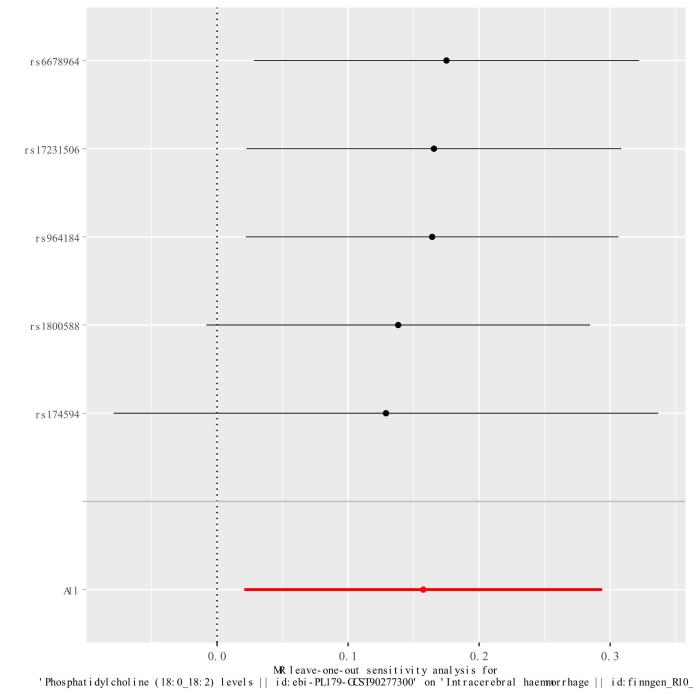

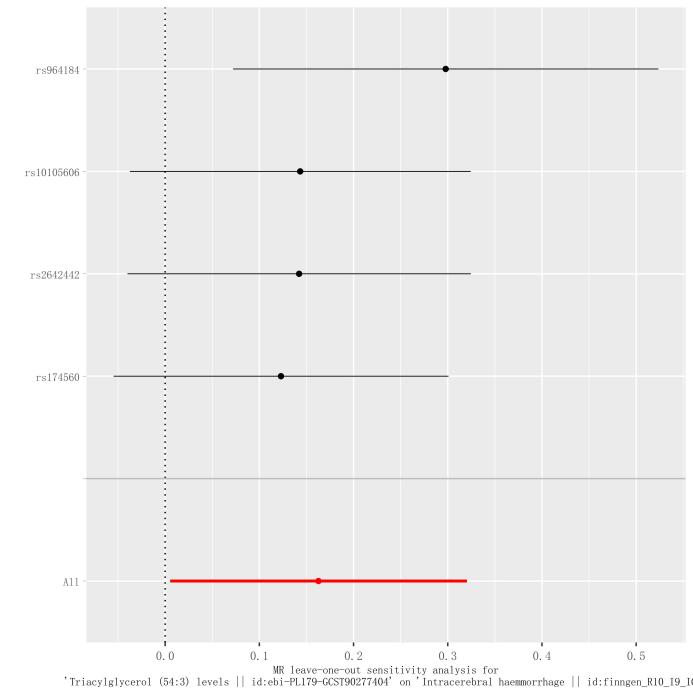

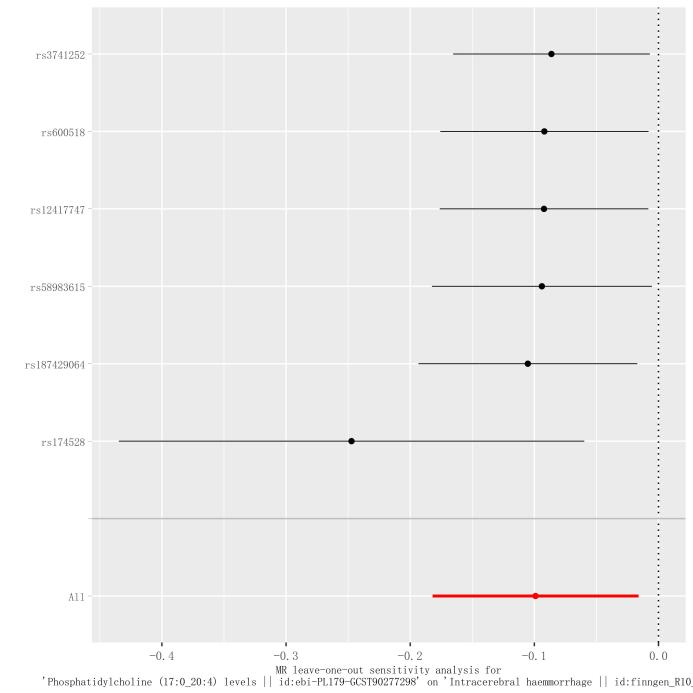

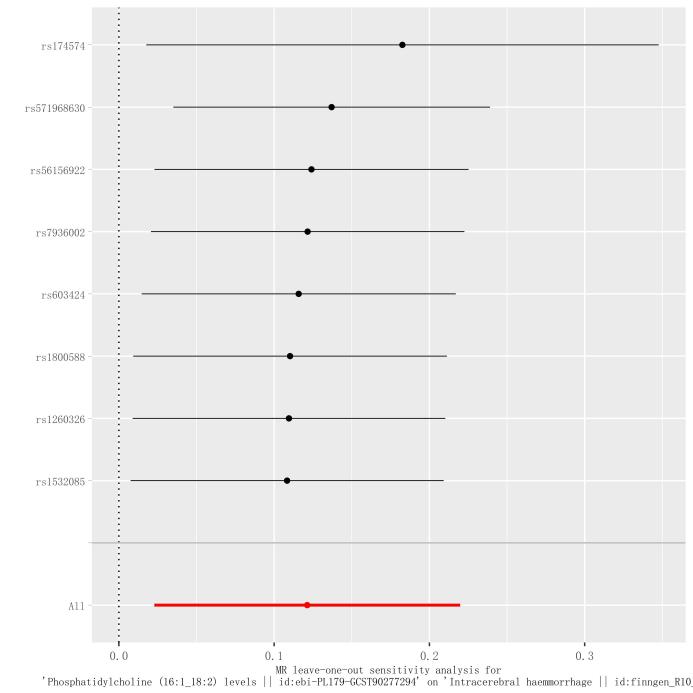

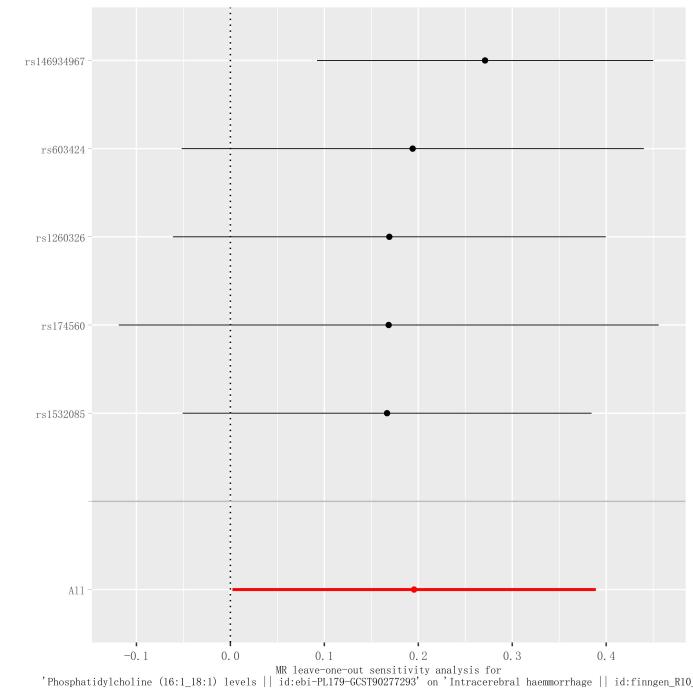

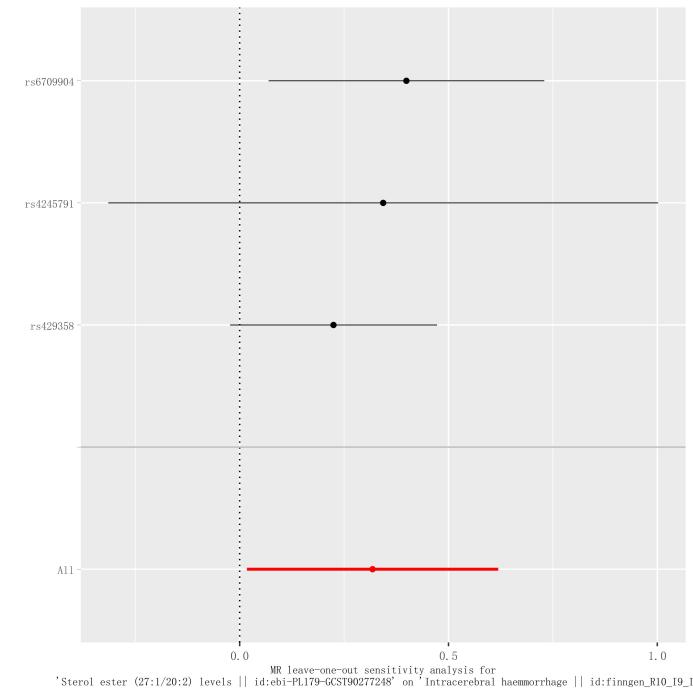


**Supplementary Figure 3.** Scatterplot of significantly associated candidate lipids with consistent directionality for the association between plasma lipids and subarachnoid hemorrhage.


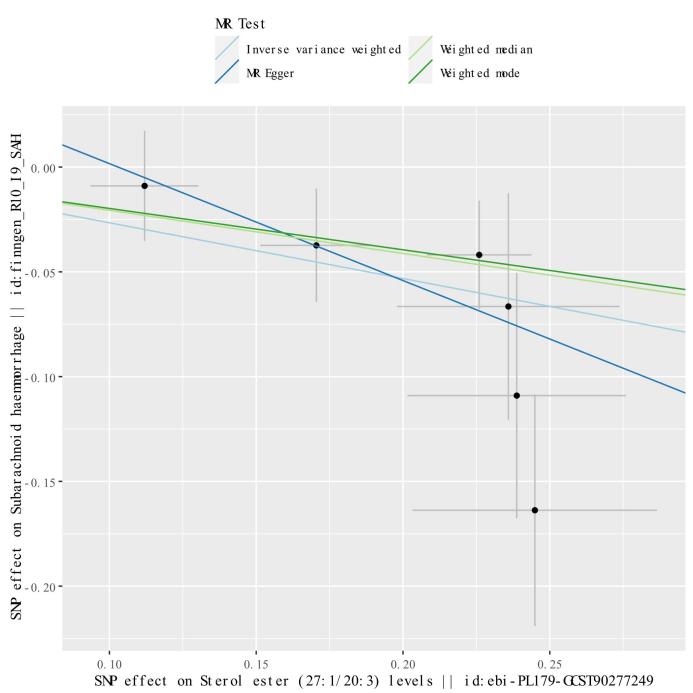

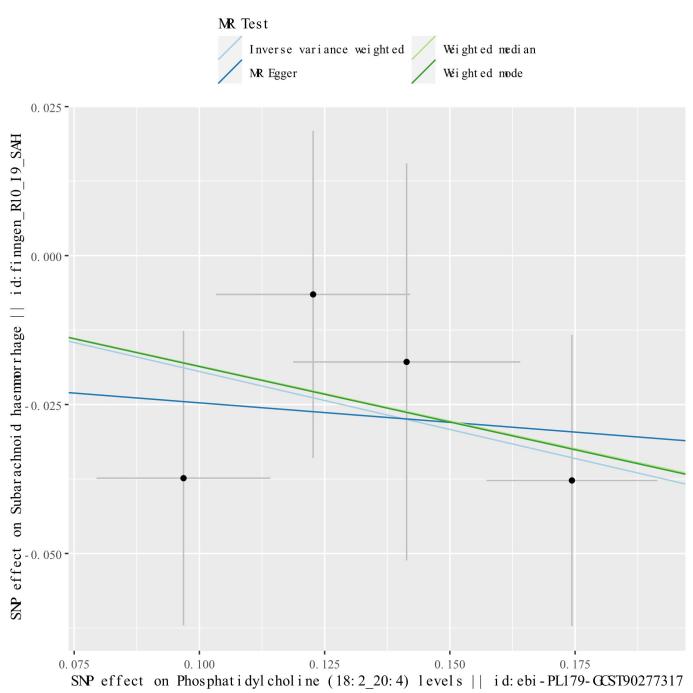

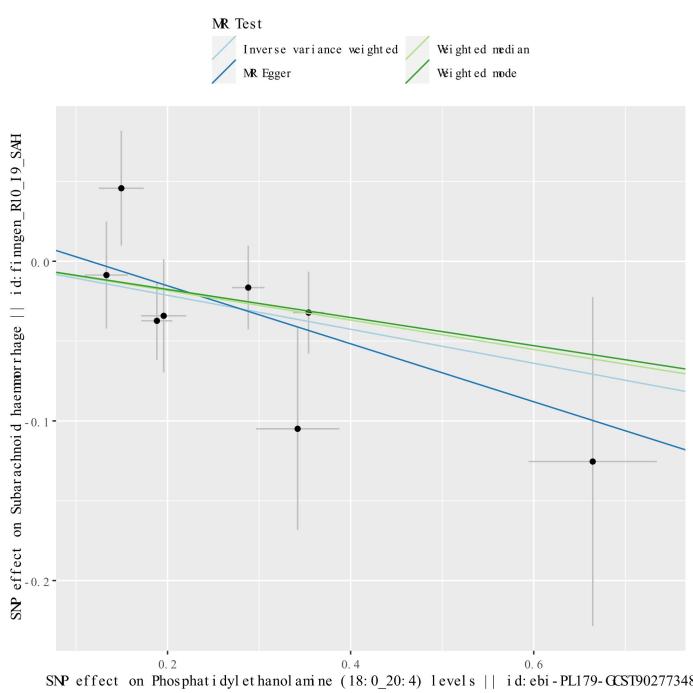

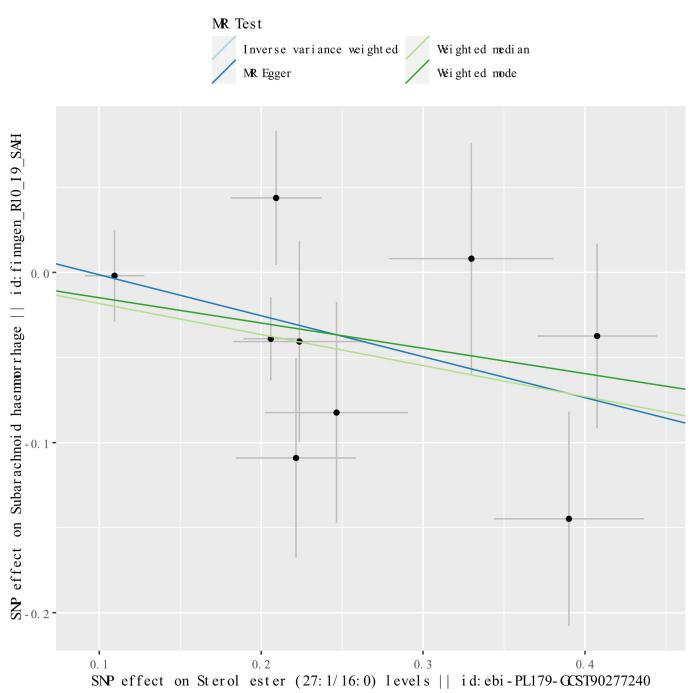

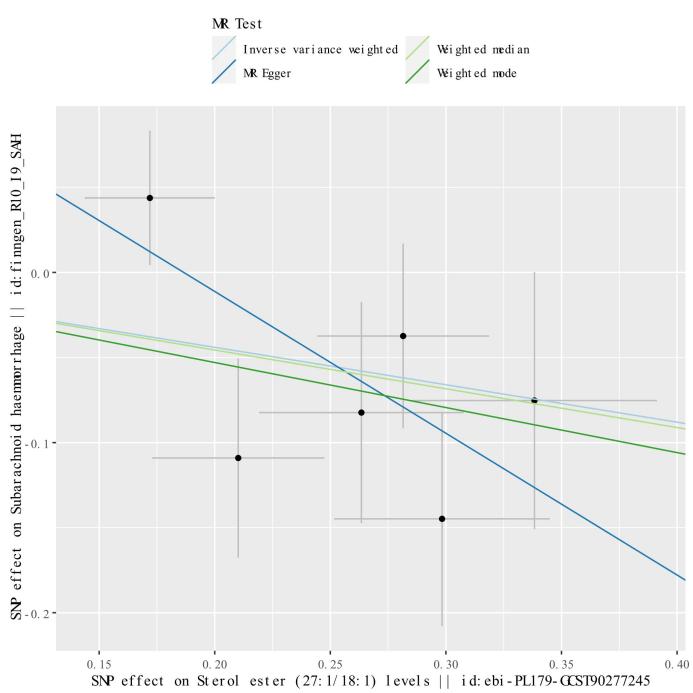

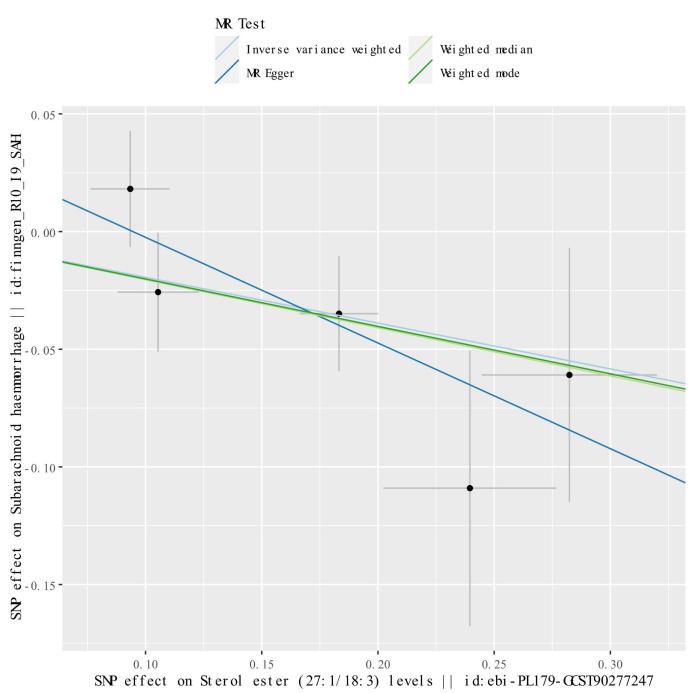


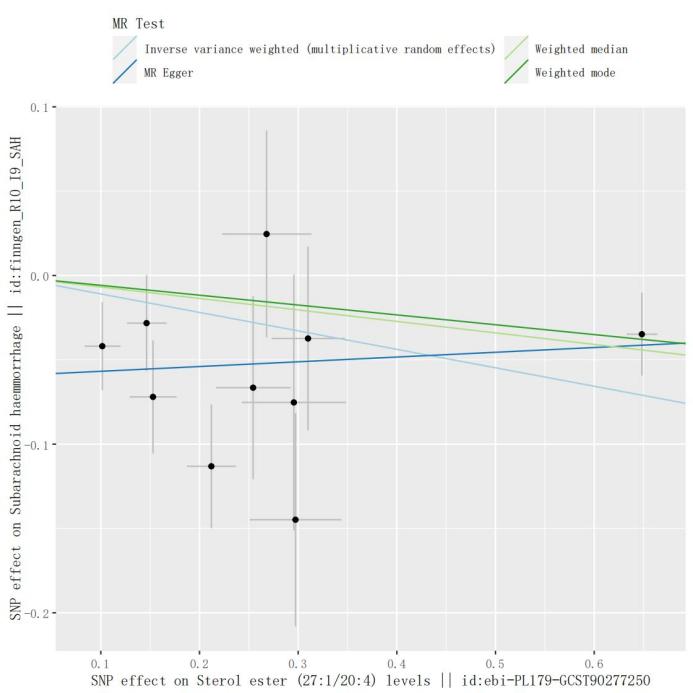

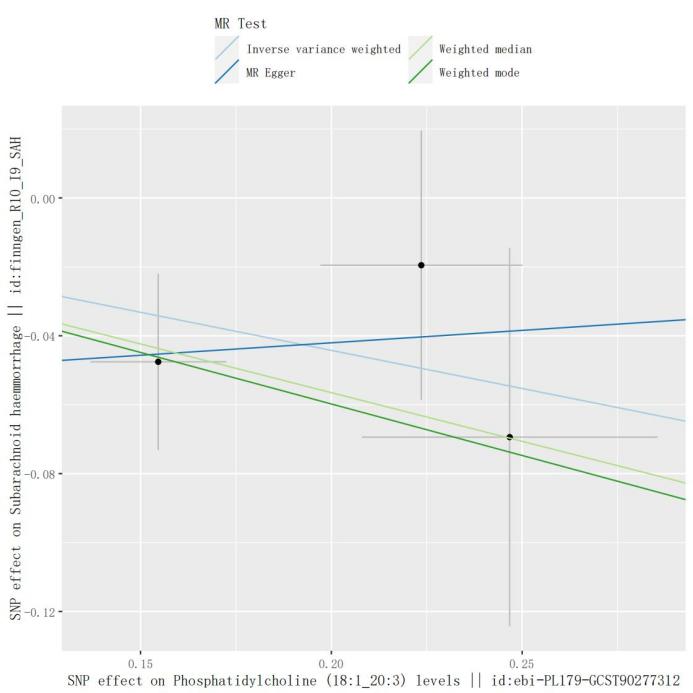


**Supplementary Figure 4.** Forest plot of leave-one-out analysis in Mendelian randomization (MR) with significant inverse variance weighted (IVW) estimates for the association between plasma lipids and subarachnoid hemorrhage.


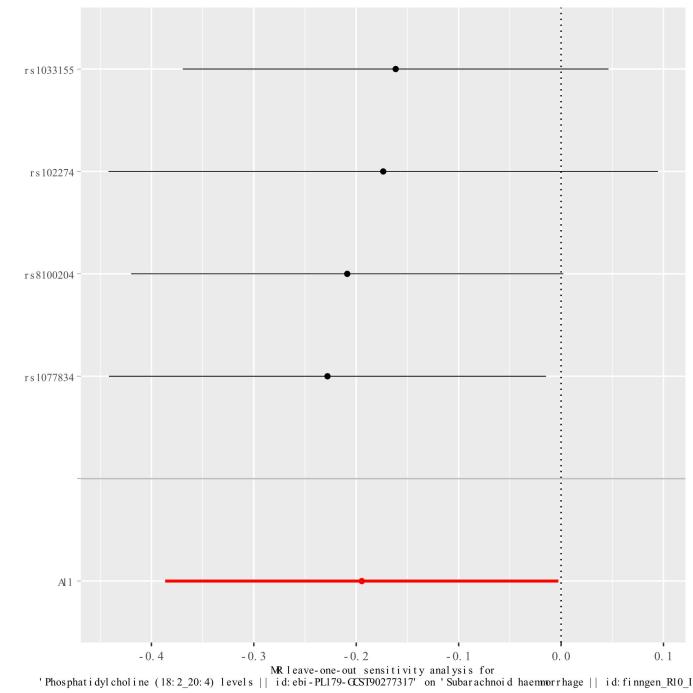

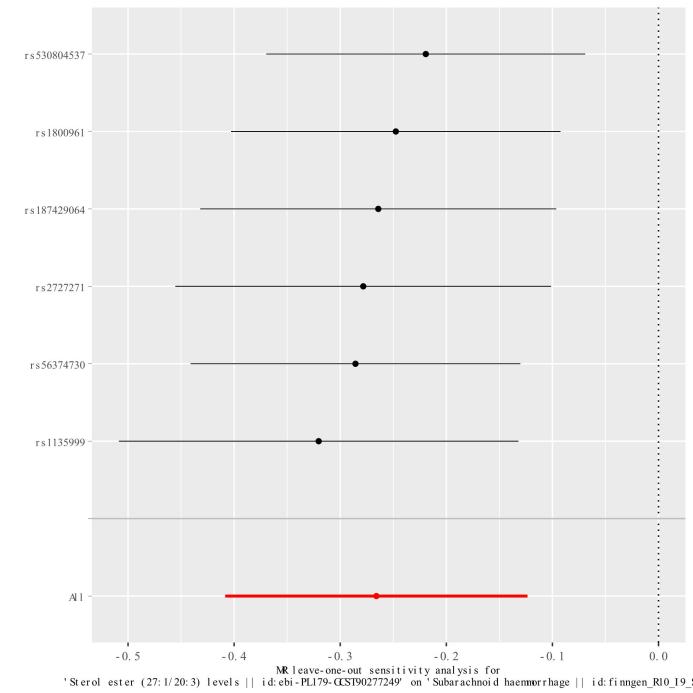

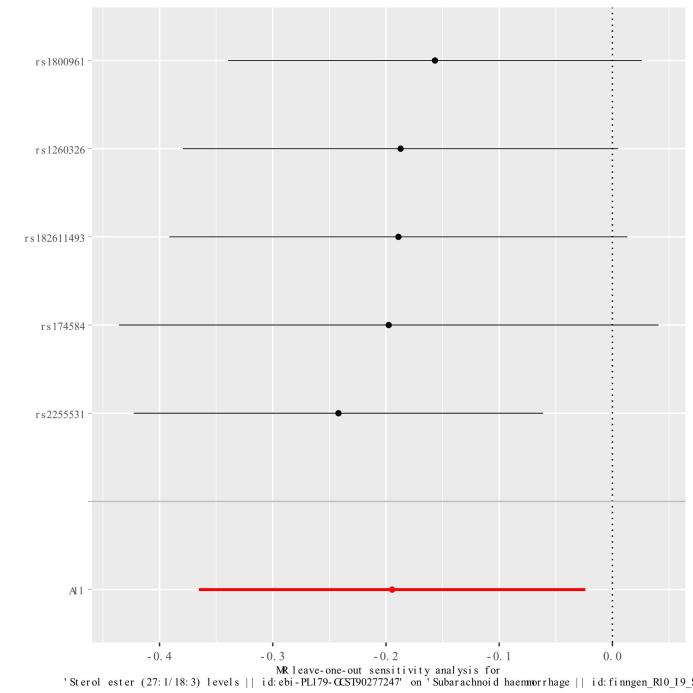

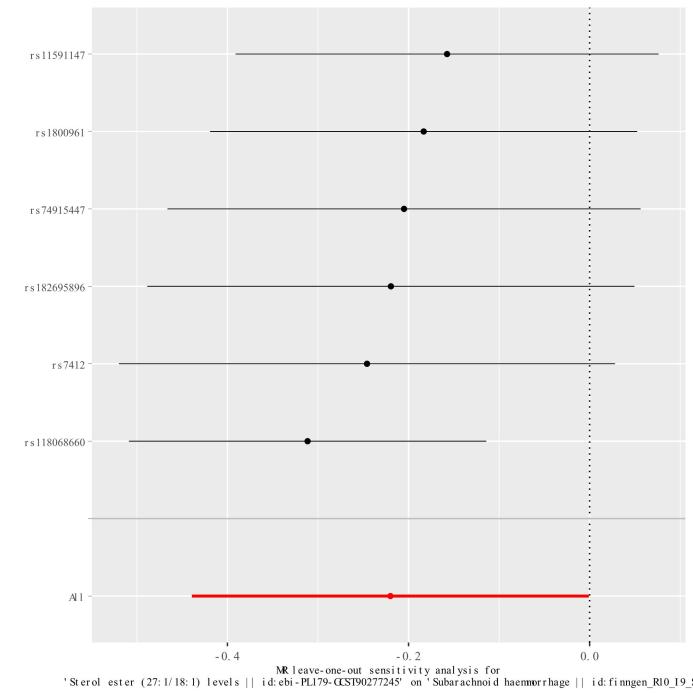

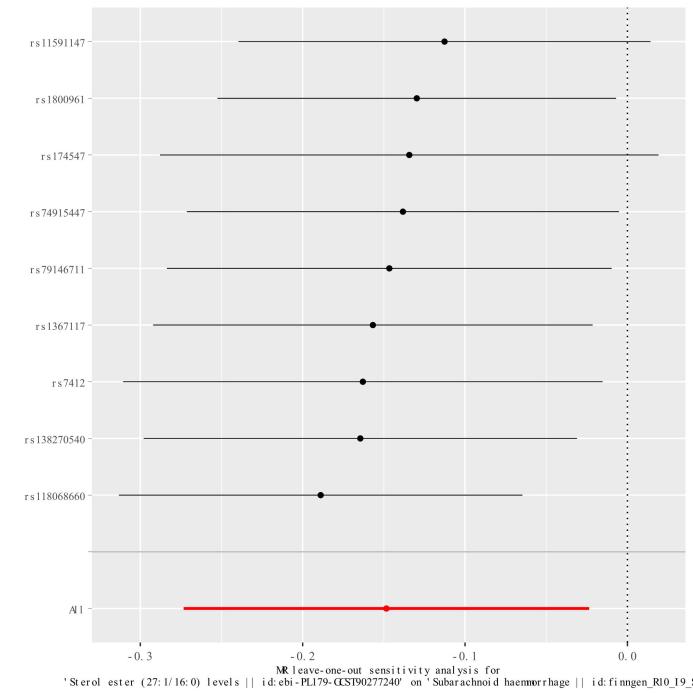

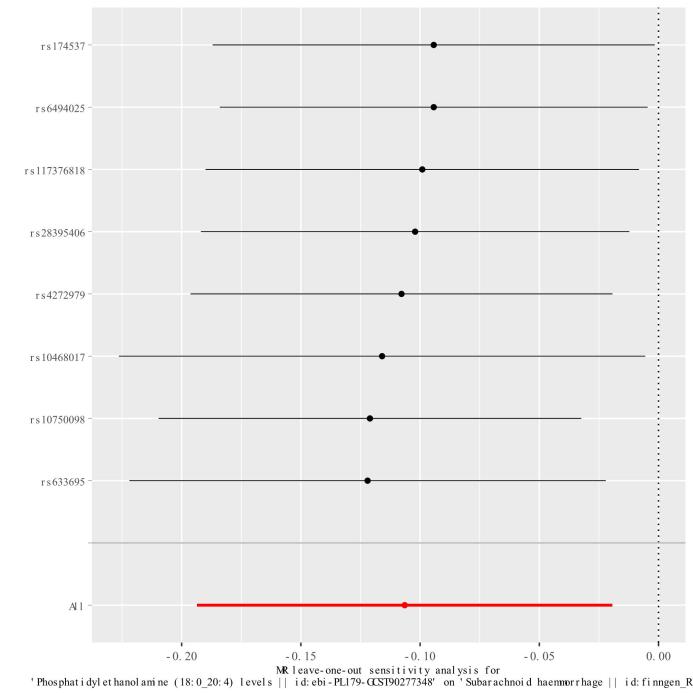


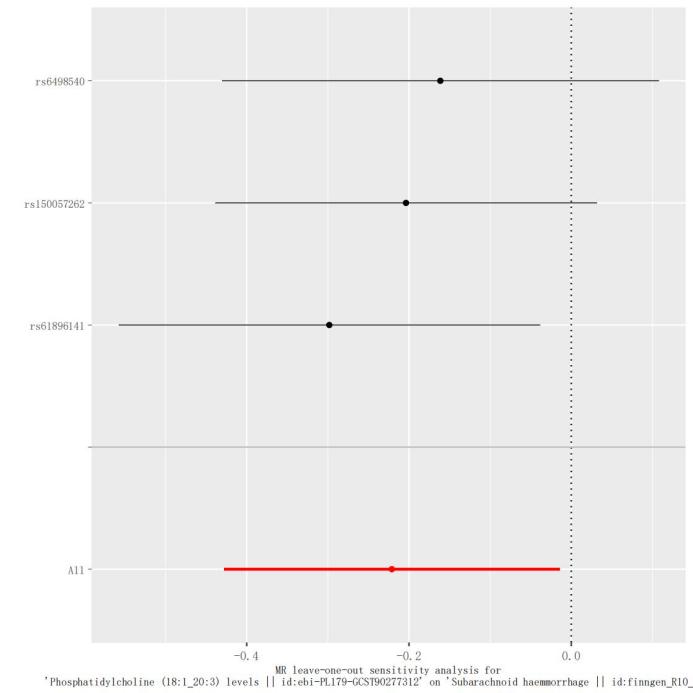

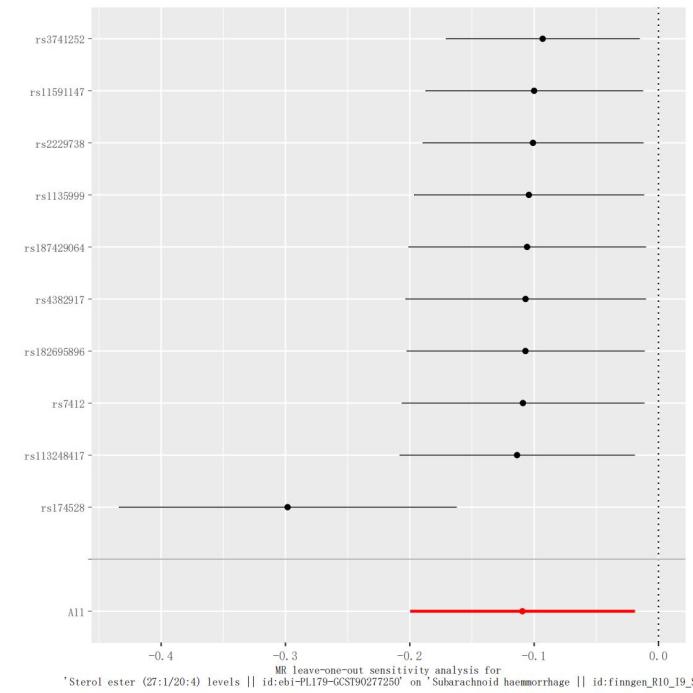


**Supplementary Figure 5**. Forest plot of MR results for plasma lipid groups carrying different fatty acid chains and hemorrhagic stroke subtypes in European populations, from Inverse variance weighted (IVW) analysis. **# ,** represents lipids with p <0.05 after FDR correction. *, represents candidate lipids identified in preliminary analysis.


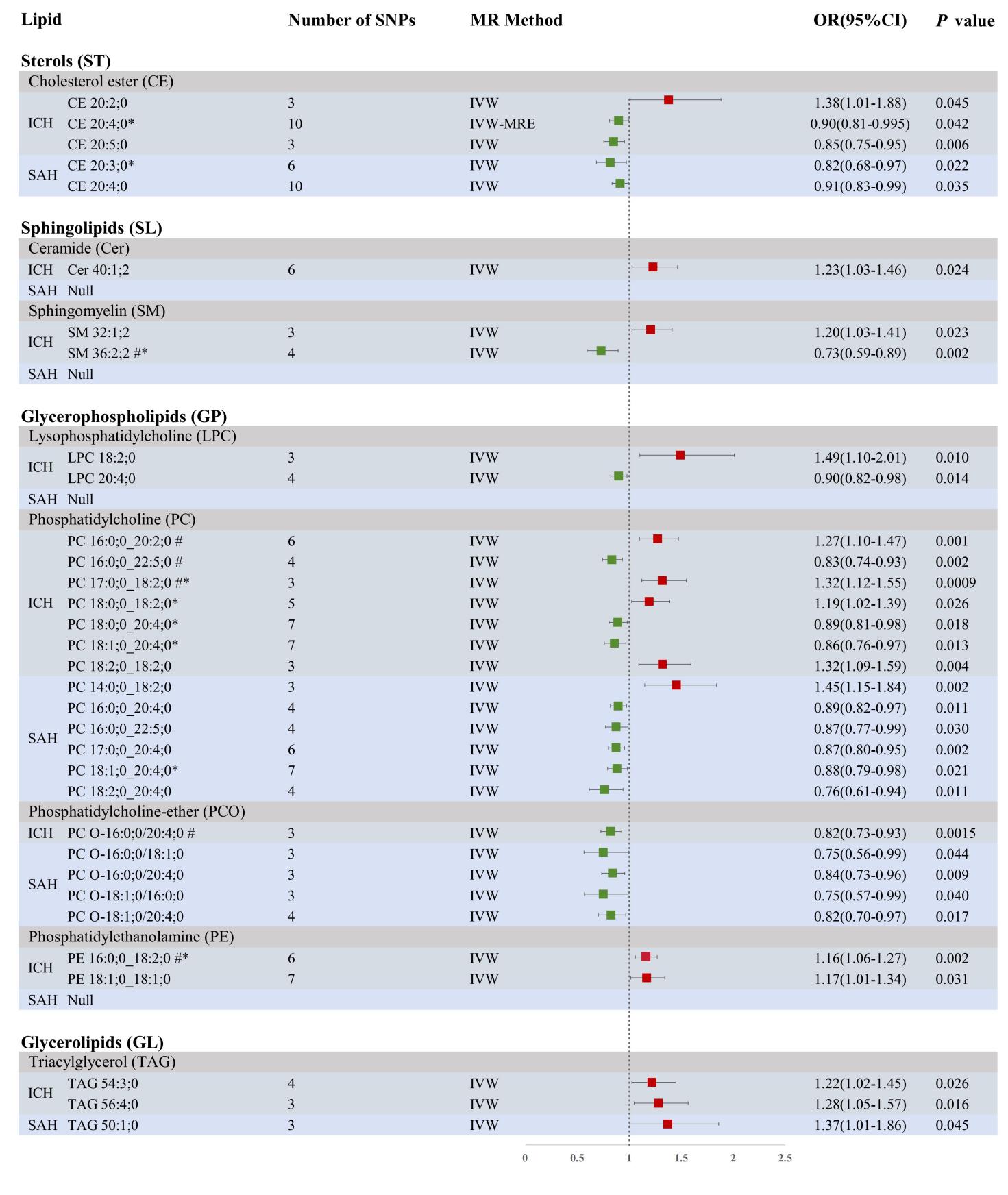

Supplement: Supplementary file 5 [file Data_Sheet_1.DOCX]
